# Genome characterization of influenza A and B viruses in New South Wales, Australia, in 2019: A retrospective study using high‐throughput whole genome sequencing

**DOI:** 10.1111/irv.13252

**Published:** 2024-01-29

**Authors:** Xinye Wang, Ki Wook Kim, Gregory Walker, Sacha Stelzer‐Braid, Matthew Scotch, William D. Rawlinson

**Affiliations:** ^1^ School of Biomedical Sciences, Faculty of Medicine and Health University of New South Wales Sydney New South Wales Australia; ^2^ Virology Research Laboratory, Serology and Virology Division (SAViD), NSW Health Pathology Prince of Wales Hospital Sydney New South Wales Australia; ^3^ Discipline of Paediatrics and Child Health, School of Clinical Medicine, Faculty of Medicine and Health University of New South Wales Sydney New South Wales Australia; ^4^ Biodesign Center for Environmental Health Engineering, Biodesign Institute Arizona State University Phoenix Arizona USA; ^5^ College of Health Solutions Arizona State University Phoenix Arizona USA; ^6^ Kirby Institute University of New South Wales Sydney New South Wales Australia

**Keywords:** drug resistance, viral, high‐throughput nucleotide sequencing, influenza, human, New South Wales

## Abstract

**Background:**

During the 2019 severe influenza season, New South Wales (NSW) experienced the highest number of cases in Australia. This study retrospectively investigated the genetic characteristics of influenza viruses circulating in NSW in 2019 and identified genetic markers related to antiviral resistance and potential virulence.

**Methods:**

The complete genomes of influenza A and B viruses were amplified using reverse transcription‐polymerase chain reaction (PCR) and sequenced with an Illumina MiSeq platform.

**Results:**

When comparing the sequencing data with the vaccine strains and reference sequences, the phylogenetic analysis revealed that most NSW A/H3N2 viruses (*n* = 68; 94%) belonged to 3C.2a1b and a minority (*n* = 4; 6%) belonged to 3C.3a. These viruses all diverged from the vaccine strain A/Switzerland/8060/2017. All A/H1N1pdm09 viruses (*n* = 20) showed genetic dissimilarity from vaccine strain A/Michigan/45/2015, with subclades 6B.1A.5 and 6B.1A.2 identified. All B/Victoria‐lineage viruses (*n* = 21) aligned with clade V1A.3, presenting triple amino acid deletions at positions 162–164 in the hemagglutinin protein, significantly diverging from the vaccine strain B/Colorado/06/2017. Multiple amino acid substitutions were also found in the internal proteins of influenza viruses, some of which have been previously reported in hospitalized influenza patients in Thailand. Notably, the oseltamivir‐resistant marker H275Y was present in one immunocompromised patient infected with A/H1N1pdm09 and the resistance‐related mutation I222V was detected in another A/H3N2‐infected patient.

**Conclusions:**

Considering antigenic drift and the constant evolution of circulating A and B strains, we believe continuous monitoring of influenza viruses in NSW via the high‐throughput sequencing approach provides timely and pivotal information for both public health surveillance and clinical treatment.

## 1 INTRODUCTION

Influenza viruses are negative‐sense, single‐stranded, segmented RNA viruses that belong to the *Orthomyxoviridae* family.^1^ Seasonal influenza, primarily caused by influenza A and B viruses (IAVs and IBVs), is a highly contagious respiratory disease that leads to significant morbidity and mortality each year.^2^ In general, seasonal influenza epidemics in humans are mainly caused by two subtypes of IAVs (A/H3N2 and A/H1N1pdm09) and two lineages of IBVs (B/Victoria and B/Yamagata). However, it is worth noting that B/Yamagata lineage viruses have not been detected since mid‐2020 worldwide. Infections with either IAVs or IBVs can give rise to serious complications, especially among high‐risk groups such as children, immunocompromised individuals, and the elderly.^3^, ^4^, ^5^

Influenza viruses, particularly IAVs, are characterized by high evolutionary rates, ranging from 2.0 × 10^−6^ to 2.0 × 10^−4^ substitutions per site per replication cycle.^2^, ^6^ Such high evolutionary rates can lead to the emergence of potential novel strain(s) and may also compromise the effectiveness of the annual influenza vaccine.^6^ While influenza vaccination is the preferred method for prevention, antiviral therapy is a vital pharmacological option for high‐risk individuals and may have the potential to reduce the risk of initial transmission.^7^, ^8^ However, mutations occurring at sites targeted by antivirals can lead to reduced or complete loss of antiviral activity. Moreover, antigenic drift adds to the uncertainty of seasonal influenza viruses from year to year. Thus, while direct phenotypic analysis is indispensable for monitoring influenza viruses, continuous genomic surveillance of circulating IAV and IBV provides supplementary insights into genetic variation and also plays an important role in public health responses.

The 2019 influenza season was one of the most severe seasons in Australia, marking a record high in the number of laboratory‐confirmed cases (*n* = 313,033). This number was approximately four times higher than the cases reported in 2018 and 2.7 times higher than the 5‐year average.^9^ New South Wales (NSW) was the hardest‐hit state during this season, accounting for nearly 37% of all confirmed influenza cases in Australia and approximately 35% of the nation's influenza‐related deaths.^10^, ^11^ Additionally, over 400 laboratory‐confirmed influenza outbreaks were reported in institutions (e.g., residential care facilities) in NSW in 2019, with the majority attributed to IAVs and a smaller portion was due to IBV outbreaks.^10^ The peak of influenza activity in NSW occurred in July of this season, which was 1 month earlier than in recent seasons, following an early rise starting from May.^11^ Despite previous studies that have analysed the epidemiology of influenza in Australia during the 2019 season based on Sanger sequencing/PCR testing results of the hemagglutinin (HA) gene,^12^, ^13^, ^14^ research assessing the genetic characteristics of influenza strains circulating in NSW specifically during this period remains limited.

This study aimed to retrospectively investigate the genetic characteristics of IAVs and IBVs circulating in NSW, Australia, in 2019 by utilizing the amplicon‐based high‐throughput sequencing (HTS) approach. The advantage of HTS over Sanger sequencing is that it enables the characterization of complete influenza genome sequences in a single run and provides comprehensive identification of variants present throughout the genome.^15^, ^16^ Additionally, this study aimed to identify known molecular markers linked to antiviral drug resistance and increased virulence by comparing the generated sequences with the vaccine strains.

## 2 METHODS

### 2.1 Clinical specimens

A total of 142 archived IAV‐ and IBV‐positive nasopharyngeal/throat/nasal swabs were collected from some patients exhibiting influenza‐like illness symptoms between July and December 2019 at the Prince of Wales Hospital and Sydney Children's Hospital in New South Wales. These samples were previously tested at NSW Health Pathology East Serology and Virology Division using the Allplex™ Respiratory Panel I/II/III assays (Seegene Inc., South Korea) and then stored at −80°C.

### 2.2 RNA extraction and RT‐qPCR

Viral RNA was extracted from 140 μL of each selected influenza‐positive specimen (*n* = 142) using a QIAamp Viral RNA Mini Kit (Cat. No. 52906, Qiagen Inc., Germany) following the manufacturer's instructions and eluted in 50 μL of nuclease‐free water. One‐step reverse transcription‐quantitative PCR (RT‐qPCR) was performed to determine the influenza viral load on a LightCycler 480 II system (Roche, Base, Switzerland) using a SensiFast Probe No‐Rox Kit (Meridian Biosciences, Australia) with specific primers and probes following published protocols.^17^, ^18^ In total, 113/142 samples (80%) with cycle threshold value (Ct values) <30 were then selected for HTS.

### 2.3 Full‐genome amplification and Illumina sequencing

The selected RNA samples were subjected to cDNA synthesis and multiplex PCR amplification using SuperScript™ III One‐Step RT‐PCR System with Platinum® Taq High Fidelity (Thermo Fisher Scientific, USA). Viral RNA segments of IAVs were simultaneously amplified using the Uni/Inf primer set (see details in Table S1) outlined in the Zhou et al. protocol.^15^ The influenza A assay was undertaken using a modified protocol, as described in Supporting Information S1. The genomic segments of IBVs were amplified simultaneously using the universal IBV‐GA2 primer cocktail, as described by Zhou et al.^16^ The amplification protocol was described in Supporting Information S1. Both PCR amplifications were performed on a SimpliAmp™ thermal cycler (Thermo Fisher Scientific, USA). Each PCR product was then purified using 0.5X Agencourt AMPure XP beads (Beckman Coulter Inc., USA). PCR amplicons were quantified using a Quant‐iT™ PicoGreen™ dsDNA Assay Kit (Thermo Fisher Scientific, USA).

Amplicon libraries for short‐read sequencing were then prepared using the Illumina DNA Prep Kit (Illumina, USA) with the Illumina DNA/RNA UD Indexes (Illumina, USA) following the manufacturer's instructions. Pair‐end sequencing was performed on the MiSeq using a MiSeq reagent kit v2 (Illumina, USA) (details in Supporting Information S1).

### 2.4 Short‐read quality and sequence assembly

Pair‐end raw reads were quality checked using the FastQC tool (v0.11.9) and then trimmed using fastp (v0.23.0) to remove low‐quality and short reads.^19^, ^20^ Clean reads were assembled using the FLU module of the Iterative Refinement Meta‐Assembler (v1.0.2) pipeline with default parameters.^21^ SAMtools (v1.15) was used to calculate the depth and coverage for each segment.^22^ The raw sequencing reads were deposited in the National Center for Biotechnology Information Sequence Read Archive database (Bioproject: PRJNA946359 [IAVs] and PRJNA946360 [IBVs]). Consensus nucleotide sequences of all segments were deposited in GenBank (accession numbers listed in Table S2).

### 2.5 Phylogenetic analysis

The generated nucleotide sequences of the HA and neuraminidase (NA) genes were trimmed of primer sequences and aligned with the reference sequences using the ClustalW program with default parameters in MEGA 11 software.^23^ Reference sequences included (1) five to eight sequences per subtype from other Australian regions (excluding NSW) and neighbouring New Zealand from May to December 2019, (2) sequences of the influenza strains (including A/Michigan/45/2015 [H1N1]‐like; A/Switzerland/8060/2017 [H3N2]‐like; and B/Colorado/06/2017‐like virus [B/Victoria lineage]) recommended for the 2019 Southern Hemisphere (SH) vaccine (egg‐propagated trivalent vaccine), and (3) clade‐defining sequences indicated in the Nextstrain platform.^24^, ^25^ All reference sequences were retrieved from the Global Initiative on Sharing All Influenza Data database (Table S3).^26^ Maximum likelihood phylogenetic analysis was performed using IQ‐Tree (v2.2.0).^27^ A/California/07/2009 (A/H1N1pdm09), A/Texas/50/2012 (A/H3N2), and B/Brisbane/60/2008 (B/Victoria) were selected as the roots for each subtype tree. ModelFinder, included in the IQ‐Tree software, was used to select the best‐fitted model based on Bayesian information criterion.^28^ The resulting HA and NA maximum likelihood trees were then edited and visualized on FigTree (v1.4.4).^29^

### 2.6 Amino acid sequence analysis

Amino acid sequences (including HA, NA, PB2, PB1, PB1‐F2, PA, NP, M1, M2, NS1, and NS2 proteins) were deduced by translating nucleotide sequences (each segment of IAVs/IBVs) using the standard genetic code in MEGA 11 software. Translated sequences were submitted to FluSurver (https://flusurver.bii.a-star.edu.sg/, accessed on 1 February 2023) to examine the amino acid substitutions compared with the egg‐propagated vaccine strains and to screen them for any phenotypically or epidemiologically interesting mutations such as mutations associated with genotypic antiviral drug susceptibility and increased virulence.

## 3 RESULTS

### 3.1 Detection of IAVs/IBVs by RT‐qPCR

In total, 112 (79%) specimens tested positive for IAV, and 30 (21%) were positive for IBV by RT‐qPCR. Ct values for these specimens ranged from 16.09 (7.75 × 10^8^ genome copies per millilitre) to 37.81 (3.71 × 10^2^ genome copies per millilitre). Following RT‐qPCR, 113 specimens with Ct values below 30 were selected for further whole‐genome sequencing.

### 3.2 Sequencing efficiency

Complete genomes of 112 of the 113 specimens were obtained. We were unable to obtain the complete genome sequence for one IAV‐positive sample but did successfully generate its partial viral genome segments (4th‐8th segments). The average number of total raw reads per sample was 614,419. On average, 97% of total reads contained high‐quality data that were ≥Q30 (quality score defined by Illumina; 1 error per 1000 sequenced bases). According to previous studies, a minimum coverage depth of 50X was required for Illumina data to be considered reliable.^30^ In this study, nearly all specimens (112/113, 99%) showed good overall sequencing depth across the entire genome (average depth >1000X per sample). Additionally, a summary of the average number of mapped reads and coverage depth for each segment of the IAV/IBV genome is provided (Table S4).

### 3.3 Sample characteristics and influenza subtyping

Sequences of 92/113 (81%) specimens were confirmed to be IAVs, with 78% (*n* = 72) identified as A/H3N2 and 22% (*n* = 20) identified as A/H1N1pdm09. The remaining 21 specimens were confirmed to be IBVs, all of which were B/Victoria lineage strains. Table 1 shows demographic characteristics and sample types. In all age groups, the A/H3N2 subtype was predominant in influenza‐infected patients, with the exception of the 5‐ to 17‐year‐old age group, where B/Victoria lineage viruses were more frequently detected (Table S5). Additionally, two IAV‐positive specimens that previously failed subtyping by diagnostic qRT‐PCR were classified as A/H3N2 by HTS.

**TABLE 1:** Characteristics of influenza‐positive (successfully sequenced) specimens collected in New South Wales, Australia.

Characteristics	*N* (%)
Gender	
Female	56 (49.6)
Male	57 (50.5)
Age group	
<5	16 (14.2)
5–17	14 (12.4)
18–49	31 (27.4)
50–64	13 (11.5)
≥65	39 (34.51)
Sample type	
Nasopharyngeal swab	103 (91.2)
Throat swab	2 (1.8)
Nose swab	8 (7.0)
IFV subtype detected	
A‐H3N1	72 (63.7)
A‐H1N1pdm09	20 (17.7)
B‐Victoria	21 (18.6)
Ward of the hospitals where received cases	
Emergency department	97 (85.8)
Oncology department	2 (1.8)
Paediatric ICU	4 (3.5)
Others	10 (8.9)

*Note*: ‘Others’ refers to the respiratory medical assessment unit, renal dialysis unit, and paediatric observation ward.

Abbreviations: ICU, intensive care unit; IFV, influenza virus.

### 3.4 Phylogenetic characterization and amino acid variation

The HA and NA gene sequences were used for phylogenetic analysis to determine the genetic relationship of NSW viruses reported in the study with vaccine viruses and viruses circulating in other Australian regions and neighbouring New Zealand during the same season. Furthermore, amino acid substitutions from each segment of IAVs and IBVs were identified as compared with the vaccine strains and were then screened for virulence markers.

#### 3.4.1 A/H1N1pdm09

**FIGURE 1:**
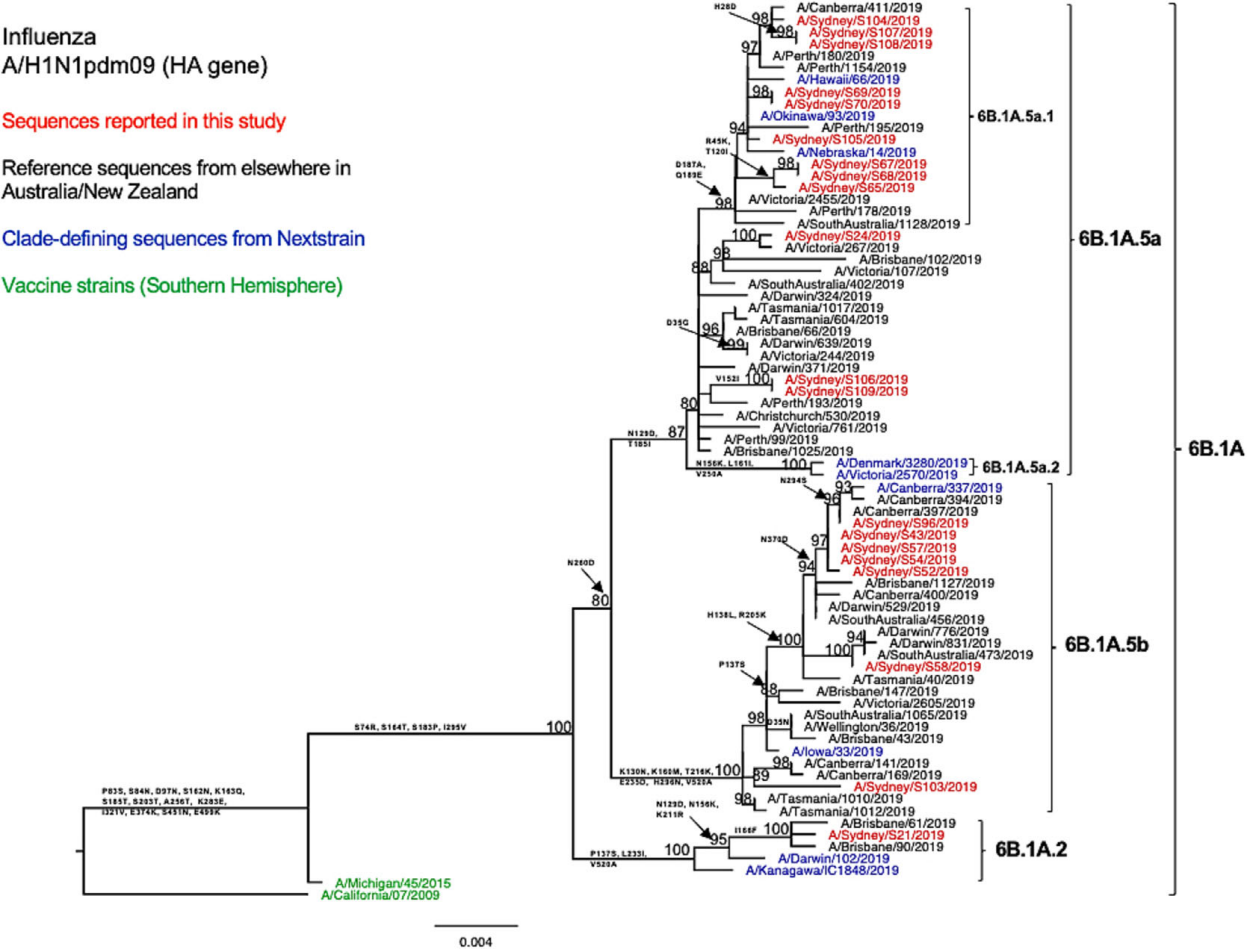
Phylogenetic analysis of the hemagglutinin (HA) gene from influenza A (H1N1pdm09) viruses detected in this study. The phylogenetic tree was constructed by the maximum likelihood method using IQ‐TREE with substitution model selection (ModelFinder implemented in IQ‐TREE) option and 1000 bootstraps. The best‐fit model according to Bayesian information criterion: HKY + F + G4 (HA). Bootstrap values are shown if >70%.

The phylogenetic tree of the HA genes showed that the 20 sequenced NSW A/H1N1pdm09 viruses fell into the clade 6B.1A, subclades 6B.1A.5 (95%, 19/20) and 6B.1A.2 (5%, 1/20) (Figure 1). Further analysis using clade‐defining sequences revealed that 9 out of 12 NSW viruses within the subgroup 6B.1A.5a could be further classified into the genetic subgroup 6B.1A.5a.1 (carried additional substitutions D187A and Q189E in HA1). Within the subclade 6B.1A5 (including subgroups 6B.1A.5a and 6B.1A.5b), other sequences reported from elsewhere in Australia and New Zealand during the 2019 season clustered together with the sequences reported in this study. Within the subclade 6B.1A.2, our sequence was closely related to viruses from Brisbane, Queensland. All these viruses were divergent from the vaccine strain (A/Michigan/45/2015), exhibiting amino acid substitutions S74R, S164T, S183P, and I295V in HA1.

**FIGURE 2:**
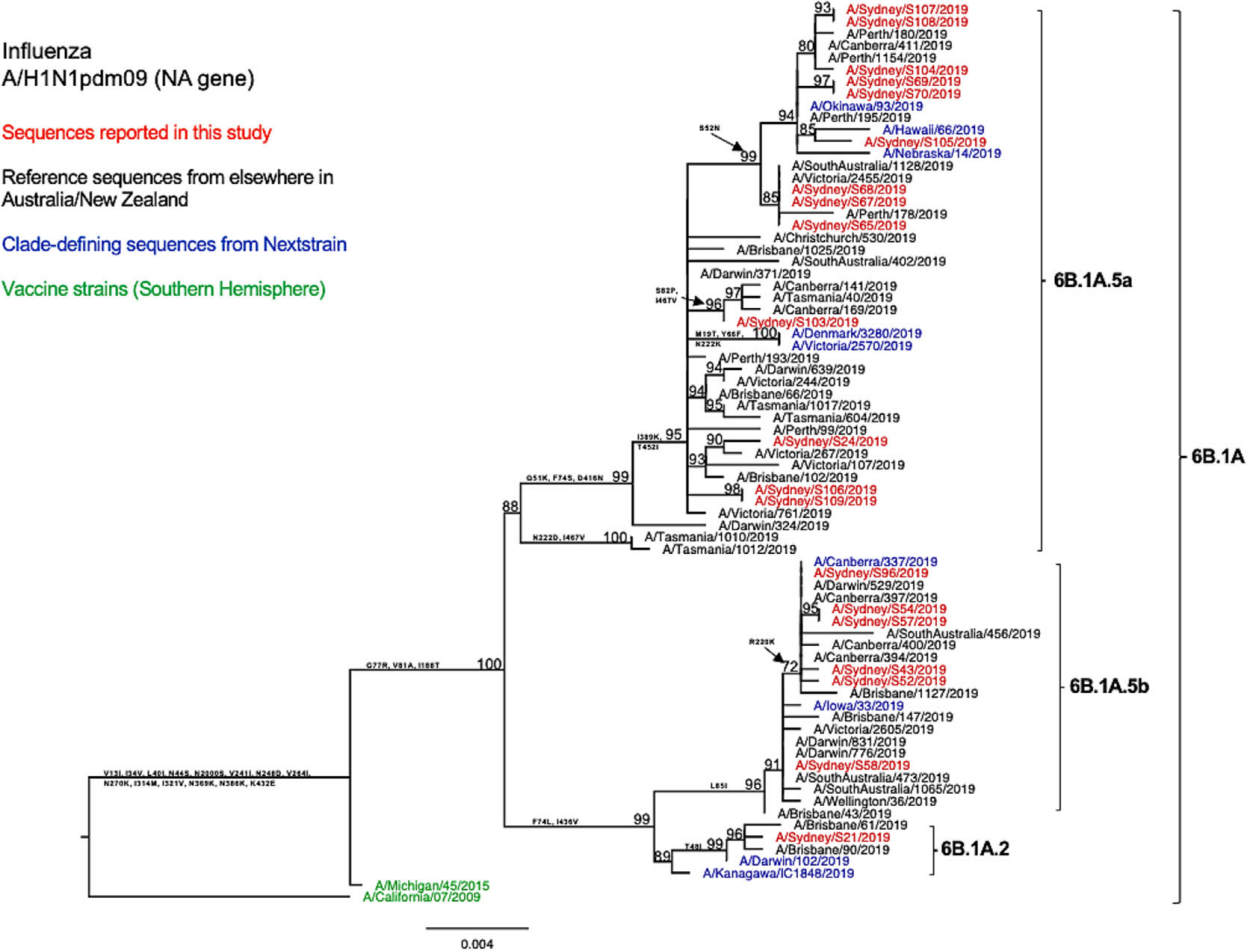
Phylogenetic analysis of the neuraminidase (NA) gene from influenza A (H1N1pdm09) viruses detected in this study. The phylogenetic tree was constructed by the maximum likelihood method using IQ‐TREE with substitution model selection (ModelFinder implemented in IQ‐TREE) option and 1000 bootstraps. The best‐fit model according to Bayesian information criterion: HKY + F + G4 (NA). Bootstrap values are shown if >70%.

In comparison with the A/Michigan/45/2015 vaccine strain, all tested HA sequences showed two shared amino acid substitutions at two of the five known antigenic sites—S74R at site Cb and S164T at site Sa.^31^ Both of these substitutions are been very common since 2017.^31^, ^32^ Among these, the S164T substitution is a modification to a glycosylation site at residues 162–164 altering Ser to Thr and probably occludes this epitope.^32^ In specific subclade/subgroups, these HA sequences exhibited additional amino acid substitutions at the known antigenic sites: Sequences (*n* = 12) within subclade 6B.1A.5a bear T185I in the Sb epitope; sequences (*n* = 9) within subgroup 6B.1A.5a.1 presented D187A and Q189E in the Sb epitope; and sequences within subclade 6B.1A.5b displayed K160M in site Sa and E235D in site Ca1. Additionally, the antigenic site Sb substitutions D187A and Q189E were found to be located at the receptor binding site (RBS).^33^ The HA amino acid sequence identity of these A/H1N1pdm viruses as compared with the vaccine strain was 97.13–98.41%. Similarly, we observed comparable genetic characteristics within our viruses when examining the NA segments (Figure 2). We also found four consistent amino acid substitutions in all tested strains when comparing our sequences to the vaccine strain for that year (Table 2).

**TABLE 2:** List of amino acid substitution differences in hemagglutinin (HA) and neuraminidase (NA) genes between 2019 southern hemisphere vaccine strains and New South Wales 2019 viruses.

Amino acid changes in HA and NA															
A/H1N1pdm09 6B.1A sequences (*n* = 20) compared with A/Michigan/45/2015															
HA	S74R (Cb)	S164T (Sa)	S183P	I295V											
NA	F74L/S	G77R	V81A	I188T											
A/H3N2 3C.2a1b sequences (*n* = 68) compared with A/Switzerland/8060/2017															
HA	E62G (E)	K92R (E)	N121K (D)	R142G (A)	N171K (D)	I406V									
NA	P126L	K220N	V303I												
A/H3N2.3C.3a sequences (*n* = 4) compared with A/Switzerland/8060/2017															
HA	H56Y	K82R	S91N (E)	N121D	A138S (A)	S144K (A)	Y159S (B)	F193S (B)	K326R	I478M	D489N				
NA	K75R	L140I	V149A	Y155H	G346V	T434A									
B/Victoria sequences (*n* = 21) compared with B/Colorado/06/2017															
HA	G129D (120‐loop)	G133R (120‐loop)	K136E (120‐loop)	164del (160‐loop)	V178I	K498R									
NA	Q371K	A395T													

*Note*: Only amino acid substitutions found in all sequences in each genetic clade are presented in this table. The amino acid numbering is counted without the signal peptide.

Amino acid sequence analysis revealed multiple substitutions in internal proteins as compared with the vaccine strain, with six amino acid substitutions in PB2, four in PB1, two in PA, four in NP, two in M2, and four in NS1 (Table S9). These mutations have not been previously found to be associated with increased virulence in A/H1N1pdm09 viruses, as indicated by FluSurver.

#### 3.4.2 A/H3N2

**FIGURE 3:**
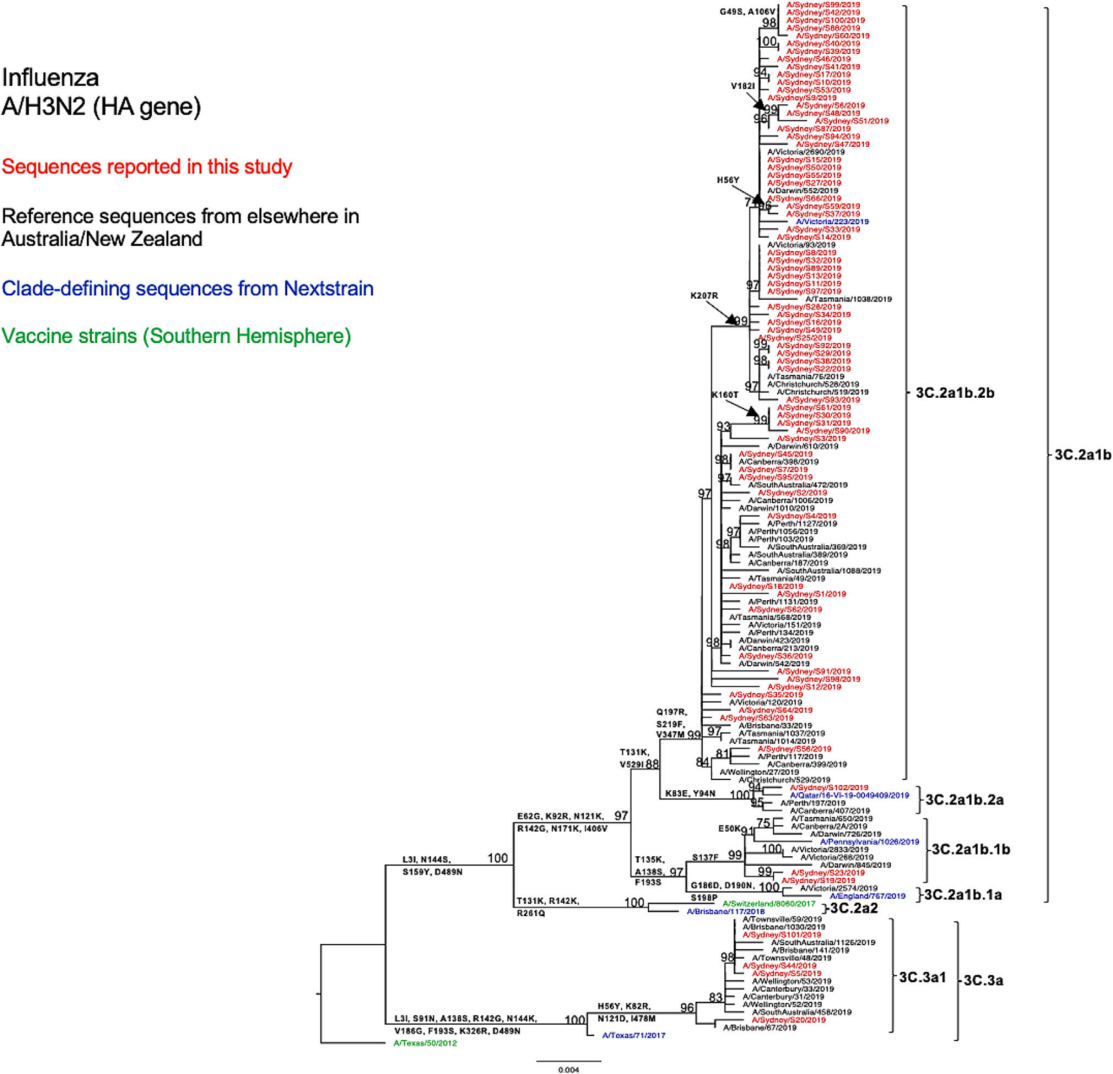
Phylogenetic analysis of the hemagglutinin (HA) gene from influenza A (H3N2) viruses detected in this study. The phylogenetic tree was constructed by the maximum likelihood method using IQ‐TREE with substitution model selection (ModelFinder implemented in IQ‐TREE) option and 1000 bootstraps. The best‐fit model according to Bayesian information criterion: TVM + F + G4 (HA). Bootstrap values are shown if >70%.

When viewing the phylogenetic structure of the HA gene, we found the NSW A/H3N2 strains (*n* = 72) divided into two genetic clades 3C.2a and 3C.3a (Figure 3). Sixty‐eight (94%) of the NSW sequences belonged to the clade 3C.2a, subclade 3C.2a1b and clustered together with the most of other sequences from elsewhere in Australia and New Zealand during the same season. Among these NSW 3C.2a1b subclade viruses, 96% fell into the subgroup 3C.2a1b.2b (shared HA1 substitutions T131K, Q197R, and S219F), 3% in the subgroup 3C.2a1b.1b (shared HA1 substitutions T135K, S137F, A138S, and F193S), and 2% in the subgroup 3C.2a1b.2a (shared HA1 substitutions K83E, Y94N, and T131K). Viruses from clade 3C.3a, specifically subclade 3C.3a1 (6%, 4/72), only constituted a small proportion of the NSW A/H3N2 viruses and were closely related to viruses reported from Brisbane and Townsville in Queensland during the same season. None of these viruses clustered with the vaccine strain A/Switzerland/8060/2017, which belonged to a different clade 3C.2a2.

**FIGURE 4:**
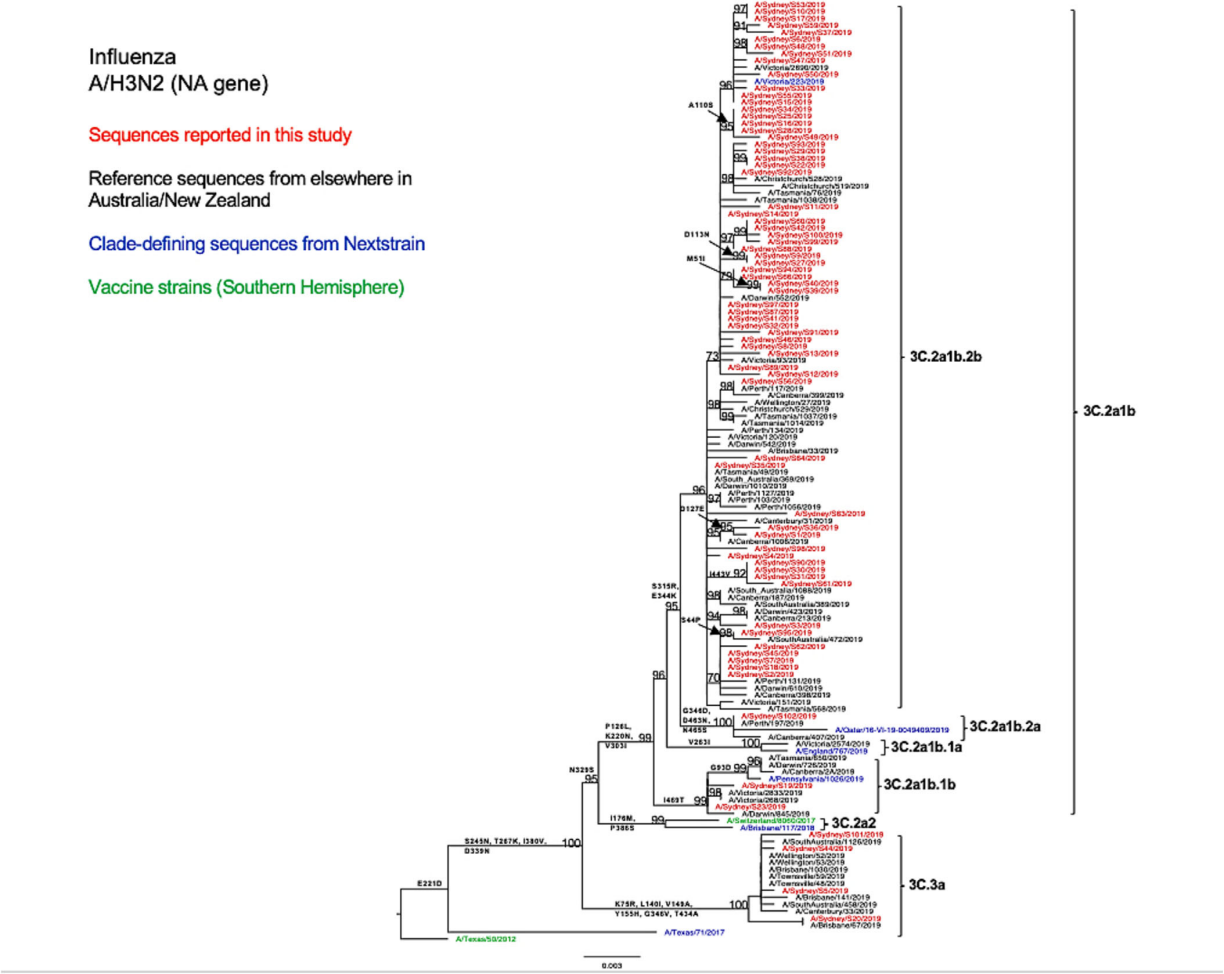
Phylogenetic analysis of the neuraminidase (NA) gene from influenza A (H3N2) viruses detected in this study. The phylogenetic tree was constructed by the maximum likelihood method using IQ‐TREE with substitution model selection (ModelFinder implemented in IQ‐TREE) option and 1000 bootstraps. The best‐fit model according to Bayesian information criterion: TVM + F + G4 (NA). Bootstrap values are shown if >70%.

Compared with the A/Switzerland/8060/2017 vaccine strain, the tested HA sequences exhibited several consistent amino acid substitutions within the known antigenic sites. Specifically, all subclade 3C.2a1b NSW viruses displayed E62G at site E, K92E at site E, N121K at site D, R142G at site A, and N171K at site D. All subclade 3C.3a NSW viruses exhibited S91N at site E, A138S at site A, S144K at site A, Y159S at site B, and F193S at site B (Table 2). The antigenic site A substitution A138S was also found to be located within the RBS.^34^ Furthermore, 3C.3a HA sequences presented more amino acid variation than 3C.2a1b HA sequences as compared with the vaccine strain (Table 2). From the comparison between the HA sequences of the A/H3N2 strains of this study and the vaccine strain of the SH for the 2019 season, we found that amino acid similarities ranged between 96.29% and 98.06% for subclade 3C.2a1b viruses and between 96.64% and 96.82% for clade 3C.3a viruses. We observed a similar genetic structure in our N2 phylogenetic tree (Figure 4) and also found several consistent amino acid substitutions as compared with the H3N2 vaccine strain for that year (Table 2).

In terms of internal proteins, the determination of amino acid substitutions among the NSW A/H3N2 strains (as compared with the vaccine strain) revealed multiple mutations in PB2, PB1, PA, NP, M2, NS1, and NS2 (Table S9). In FluSurver, none of the known mutations associated with increased virulence in A/H3N2 viruses were detected.

#### 3.4.3 B/Victoria

**FIGURE 5:**
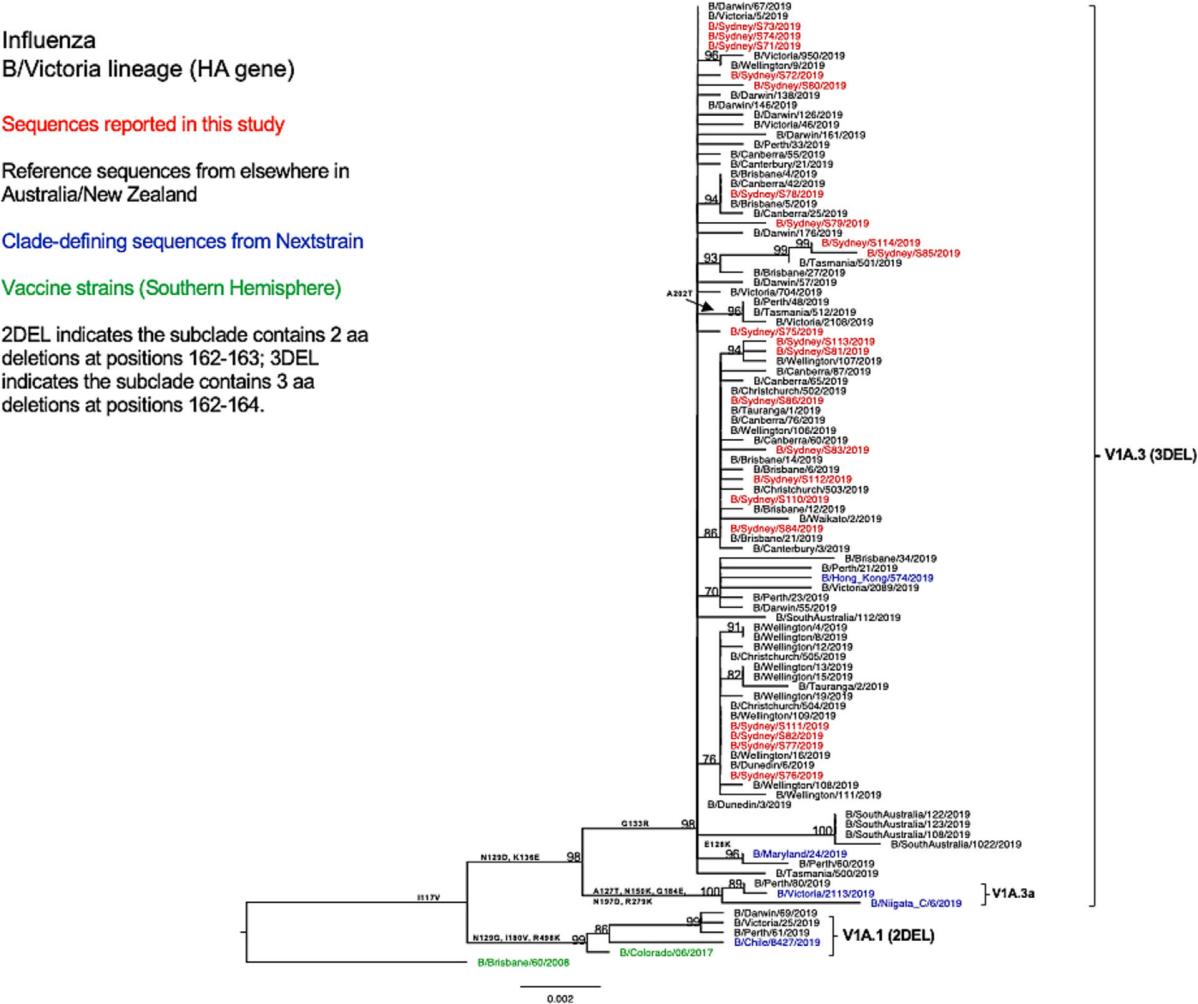
Phylogenetic analysis of the hemagglutinin (HA) gene from influenza B (Victoria lineage) viruses detected in this study. The phylogenetic tree was constructed by the maximum likelihood method using IQ‐TREE with substitution model selection (ModelFinder implemented in IQ‐TREE) option and 1000 bootstraps. The best‐fit model according to Bayesian information criterion: K3Pu + F + G4 (HA). Bootstrap values are shown if >70%.

Phylogenetic analysis of the HA gene (Figure 5) revealed that all NSW B/Victoria lineage viruses (*n* = 21) under this study fell into the clade V1A, subclade V1A.3 (characterized by triple amino acid deletions in HA1 at positions 162–164). These NSW sequences clustered together with the majority of sequences reported from other Australian regions, as well as all sequences reported from New Zealand, with shared amino acid substitutions of I117V, N129D, G133R, and K136E in HA1. Only a few viruses from Western Australia, Victoria, and the Northern Territory were classified into the subclade V1A.1 (with double amino acid deletions at positions 162–163 in HA1) and clustered with the vaccine strain recommended for SH in the 2019 season (B/Colorado/06/2017).

**FIGURE 6:**
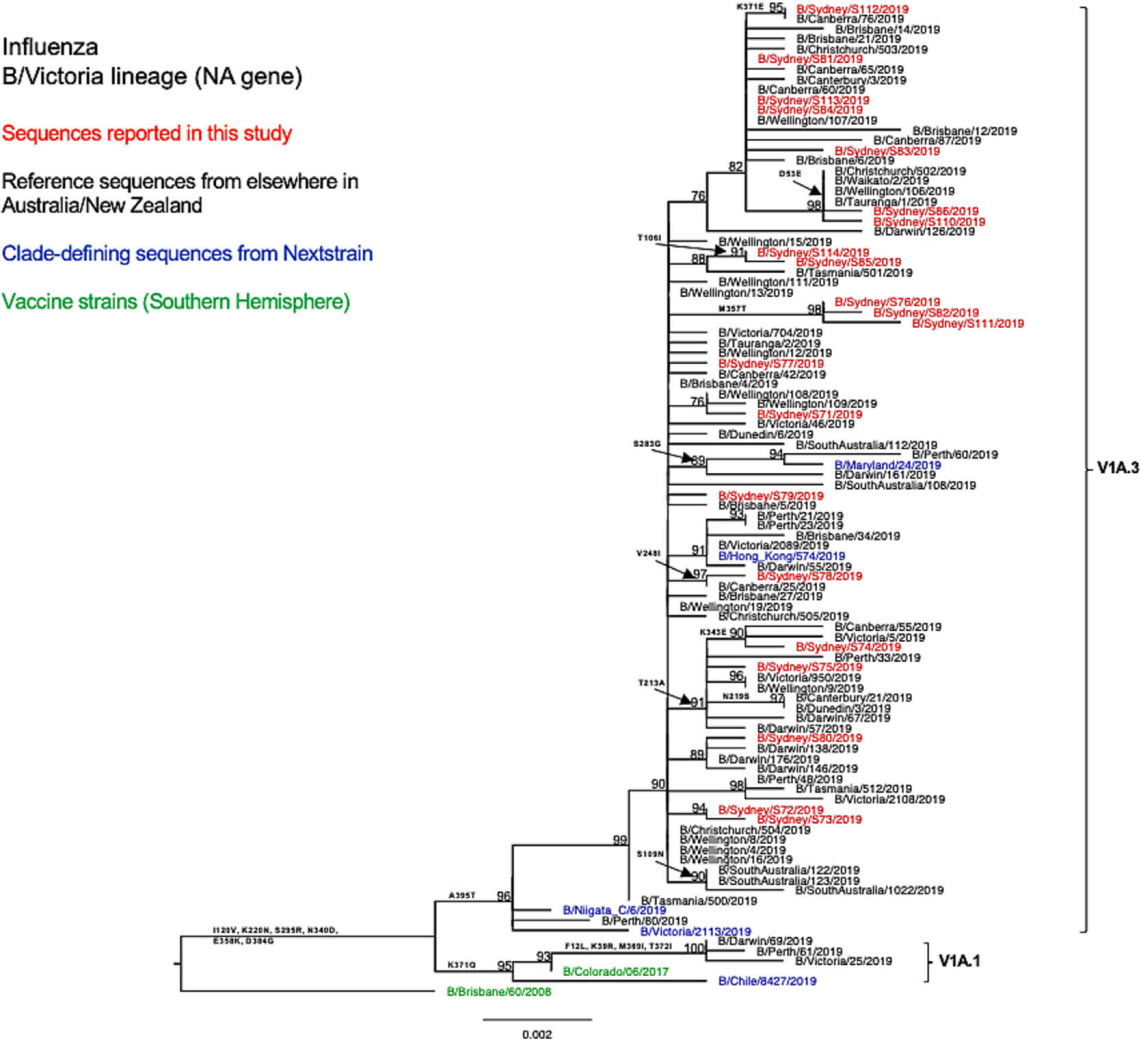
Phylogenetic analysis of the neuraminidase (NA) gene from influenza B (Victoria lineage) viruses detected in this study. The phylogenetic tree was constructed by the maximum likelihood method using IQ‐TREE with substitution model selection (ModelFinder implemented in IQ‐TREE) option and 1000 bootstraps. The best‐fit model according to Bayesian information criterion: HKY + F (NA). Bootstrap values are shown if >70%.

As compared with the vaccine strain B/Colorado/06/2017, all 21 samples reported in this study exhibited substitutions in the known antigenic sites: G129D, G133R, and K136E in the 120‐loop area and 164del in the 160‐loop area (Table 2). Further analysis of these NSW B/Victoria lineages viruses showed that their similarity to the vaccine strain at the HA amino acid level ranged from 98.63% to 98.8%. A similar clade classification was observed in the NA phylogenetic tree of B/Victoria‐lineage viruses (Figure 6). We also found two consistent mutations in all NA genes when compared with the vaccine strain (Table 2).

Multiple consistent amino acid substitutions were detected in internal proteins compared with those of the 2019 vaccine virus, including I208V for the PB1 segment, E311G, E329G, and C387F for the PA segment, V453A, V481I, and M374I for the NP segment, and four substitutions for the NS2 segment (Table S9). In FluSurver, no known mutations associated with increased virulence in B/Victoria lineage viruses were detected.

### 3.5 Genotypic antiviral drug susceptibility

Sequence analysis of the NA gene revealed that one A/H1N1pdm09 virus (subclade 6B.1A.5) (1/20; 5%) contained the amino acid substitution H275Y (see Table S6). This substitution has been documented to confer highly reduced sensitivity to oseltamivir (NA inhibitors [NAIs]) in A/H1N1pdm09 viruses.^35^ Moreover, one A/H3N2 virus (subclade 3C.3a.1) was found (1/72; 1.39%) to harbour the NA substitution I222V (Table S6). Resistance mutations to M2 protein inhibitors were identified. All A/H3N2 and A/H1N1 viruses contained the S31N substitutions in their M2 proteins (Table S7). Among these S31N‐containing viruses, four A/H3N2 viruses harboured the V27I mutation, and two A/H1N1 viruses harboured the V27A mutation. The combinations S31N + V27I/A have been also shown to retain their drug‐resistance properties.^36^ None of the influenza viruses sequenced in this study had the substitution I38T in their PA sequences that were associated with reduced susceptibility to baloxavir (Table S8).^37^

## 4 DISCUSSION

Using amplicon‐based HTS, we identified influenza A/H3N2, A/H1N1pdm09, and B/Victoria lineage viruses from archived clinical specimens collected in the 2019 influenza season in NSW, similar to those observed in other regions of Australia and certain other countries, with the A/H3N2 subtype predominating.^32^, ^38^, ^39^ This is a significant change from the previous 2018 season in NSW, where the A/H1N1 viruses were more prevalent than the A/H3N2 viruses.^40^

Predominantly, the NSW A/H3N2 viruses under this study fell into the clade 3C.2a1b, with a few viruses in the clade 3C.3a. Within clade 3C.2a1b, most NSW A/H3N2 viruses were further classified into the subclade 3C.2a1b + T131K, harbouring additional amino acid substitutions Q197R, S219F, V347M, and V529I in the HA protein. However, this subclade was in the minority among A/H3N2 viruses that circulated in Australia in 2018.^13^ Two NSW sequences in the subclade 3C.2a1b + T135K carried additional amino acid substitutions S137F, A138S, and F193S in the HA protein. These additional substitutions were not reported in 2018 but became common in China and Bangladesh in 2019.^41^ NSW A/H3N2 viruses exhibited multiple mutations across antigenic sites (A, B, D, and E), potentially affecting vaccine efficacy. In addition, a report indicated that the early vaccine effectiveness estimate in Australia in 2019 was only 37% (95% confidence interval (CI): 24–49).^42^ Phylogenetic analysis further revealed that these NSW sequences were divergent from the vaccine sequence A/Switzerland/8060/2017, suggesting a low match between the vaccine strain and these NSW viruses. This divergence was also corroborated by virus neutralization assay results reported for subclades 3C.2a1b and 3C.3a viruses.^43^ Collectively, these findings may, in part, account for the upsurge in H3N2 infections observed in NSW during the 2019 season.

Most A/H1N1pdm09 viruses circulating in NSW, as identified in our study, belonged to the subclade 6B.1A.5, and few were assigned as 6B.1A.2. About half of the subclade 6B.1A.5 viruses detected in NSW presented additional amino acid substitutions D187A and Q189E (located within antigenic site Sb), which were not reported in Australia's previous season. Interestingly, these variants became more prevalent in Northern Hemisphere countries during the 2019–2020 winter season.^38^, ^44^, ^45^ Additionally, this subclade was shown to have more substitutions in its antigenic regions compared with other subclades.^32^, ^38^, ^44^, ^46^ Despite these identified viruses harboured multiple additional amino acid substitutions compared with the 2019 vaccine strain, these did not seem to have a significant impact on vaccine effectiveness, which was estimated at 62% (95% CI: 39–78) in Australia in 2019.^42^ Nevertheless, it should be noted that the World Health Organization (WHO) observed a reduction in hemagglutination inhibition (HI) titres in many recent A/H1N1pdm09 viruses that contain the HA1 amino acid substitution S183P compared with both cell culture‐propagated vaccine virus and egg‐propagated vaccine virus (A/Michigan/45/2015).^43^ Consequently, the 2020 vaccine strain for SH was updated to A/Brisbane/02/2018. Furthermore, the amino acid substitutions detected at antigenic sites (Sa and Cb) in all NSW A/H1N1pdm09 viruses were similar to those found in viruses from Thailand and China in 2019, but their impact on virus fitness remains uncertain.^47^, ^48^ The HA1 substitution S183P detected in these A/H1N1pdm09 viruses has also been reported to be near RBS which may enhance receptor binding affinity.^49^

All B/Victoria strains identified in NSW in 2019 were of the genetic subclade V1A.3, exhibiting a triple deletion of HA1 residues 162–164. This subclade emerged in 2018 and subsequently became dominant globally in 2019.^32^, ^50^ These NSW B/Victoria strains formed a distinct cluster separate from the 2019 vaccine strain in the clade V1A.2, indicating their genetic divergence from the vaccine strain B/Colorado/06/2017. The HI test result reported by WHO corroborated these findings, suggesting the antigenic change in V1A.3 viruses compared with the corresponding vaccine strain.^43^ This subsequently prompted the update of the vaccine strain to B/Washington/02/2019 for the 2020 SH season.

NAI resistance‐related mutations were detected in two patient samples. One oncology patient was infected with A/H1N1pdm09 carrying the oseltamivir‐resistant mutation H275Y. This mutation has been reported to be present in 0.8% of recently circulating A/H1N1pdm09 viruses in Asia–Pacific regions, mainly found in immunocompromised patients.^51^ However, a lack of patient clinical records makes it unclear whether this mutation occurred before or after NAI treatment. Another NA substitution I222V was detected in a patient with A/H3N2. This substitution has been shown to reduce the oseltamivir active site hydrophobicity but increase resistance to oseltamivir when it co‐occurred with the mutation E119V in A/H3N2 viruses.^52^ No NAI‐resistance markers were found in B/Victoria‐lineage viruses, which is consistent with the low frequency (<1%) of the resistant mutations in IBVs reported globally.^53^

Baloxavir marboxil (Xofluza), a cap‐dependent endonuclease inhibitor, has been approved for the treatment of uncomplicated influenza in Australian patients aged 12 and over.^54^ However, during the 2018–2019 influenza season in Japan, the mutation I38T identified in 2.3% of A/H1N1pdm09 viruses and 8% of A/H3N2 viruses^55^ was shown to reduce susceptibility to baloxavir marboxil.^37^ Our genotypic screening found no evidence of this variant in all influenza virus strains tested, suggesting that Xofluza remains an effective treatment alternative to NAIs in Australia. Several novel antiviral drugs targeting different influenza viral proteins (including PB2, PB1, PA, M2, and NA) are under development.^56^, ^57^, ^58^ With the increased use of diverse antiviral drugs in the future, it is expected that more drug‐resistant mutations may emerge across the entire genome. Therefore, the importance of adopting advanced genomic monitoring techniques, such as HTS, is highlighted. Continuous genotypic surveillance of antiviral sensitivity in clinical influenza virus isolates is essential for guiding timely clinical interventions. Further, immunocompromised patients have been reported to shed the virus longer, potentially leading to treatment failure or elevated transmission rates.^52^ These patients may shed resistant strains while remaining infectious can potentially facilitate the spread of resistant strains in the community.^51^ Therefore, vigilant monitoring of influenza‐infected immunocompromised patients in hospitals is crucial for identifying resistant strains of influenza viruses and informing early prevention strategies.

Multiple amino acid substitutions were identified in the internal proteins of IAVs and IBVs, but their functional consequences are currently unclear. No known virulence markers were detected in any of the examined IAVs (A/H1N1pdm09: D222G/E in HA, T588I, E627K, and D701N in PB2, and R108K and I123V in NS1; A/H3N2: K615E in PA) and IBVs (K338R in PA).^59^, ^60^, ^61^, ^62^ Some amino acid substitutions found in A/H1N1pdm09 (e.g., G225S and V667I in PB2, V200I and K386R in PB1, and T80A and A155T in NS1), A/H3N2 (e.g., S107N in PB2 and K158R in PA), and B/Victoria lineage viruses (e.g., V453A in NP) were similar to those found in hospitalized patients in Thailand during the 2018–2019 influenza season.^47^ However, further investigation is needed to determine if these mutations have an impact on the virulence of the influenza viruses.

This study is not without limitations. First, there were no available data on the antigenic characteristics of these NSW influenza viruses, so our analysis was limited to their genotypic characteristics. However, the antigenic analysis of 2019 influenza viruses provided by WHO can complement our study and speculate that the A/H3N2, A/H1N1pdm09, and B/Victoria viruses identified in NSW are antigenically different from the corresponding vaccine strains.^43^ Second, our results should be interpreted with caution given the small sample size and restricted geographic scope. Despite these limitations, we have provided additional insights into the genetic characteristics and whole‐genome amino acid variation of influenza viruses prevalent among patients from NSW state specifically. All these findings, in conjunction with the existing Global Initiative on Sharing All Influenza Data sequence data, can serve as a reference for future influenza research.

## 5 CONCLUSION

As the most populous state in Australia, NSW was perceived to play a significant role in the interstate and interterritorial transmission of influenza viruses. Given the pandemic potential of influenza viruses, it is crucial to consistently monitor for antigenic drift and drug resistance in circulating influenza strains in NSW via HTS. Such surveillance is key to facilitating the early detection of emergent strains and providing vital information to other Australian states and territories.

## AUTHOR CONTRIBUTIONS

**Xinye Wang:** Conceptualization; formal analysis; investigation; methodology; visualization; writing—original draft. **Ki Wook Kim:** Writing—review and editing. **Gregory Walker:** Writing—review and editing. **Sacha Stelzer‐Braid:** Writing—review and editing. **Matthew Scotch:** Conceptualization; formal analysis; methodology; supervision; writing—review and editing. **William D. Rawlinson:** Conceptualization; supervision; writing—review and editing.

## CONFLICT OF INTEREST STATEMENT

The authors declare no conflict of interest.

## ETHICS STATEMENT

Ethical approval was received from the Sydney Children's Hospitals Network Human Research Ethics Committee (ETH02051).

## Supporting information


**Table S1.** List of Primers for Multiplex RT‐PCR Amplification of Influenza A and B Viruses
**Table S2.** List of accession number (GenBank) of each segment of studied influenza viruses
**Table S3.** List of Reference Sequences Downloaded from GISAID
**Table S4.** Summary Table of Average Number of Mapped Reads and Average Coverage Depth for Study Samples
**Table S5.** Distribution of influenza virus subtypes by age groups from our study specimens between July and December of 2019 in NSW, Australia
**Table S6.** Identification of amino acid mutations associated with antiviral drug (NAIs) resistance ‐ seasonal influenza viruses
**Table S7**. Identification of amino acid mutations related to antiviral drug (M2) resistance ‐ seasonal influenza viruses
**Table S8.** Identification of amino acid mutation related to antiviral drug (Baloxavir) resistance ‐ seasonal influenza viruses
**Table S9**. List of amino acid differences in the internal proteins between NSW 2019 viruses and the vaccine strains in the same season

## Data Availability

All data generated during this study are contained within this manuscript and its supporting information files.
