# Supplementary material for: Genome characterization of influenza A and B viruses in New South Wales, Australia, in 2019: A retrospective study using high‐throughput whole genome sequencing
Source: Influenza Other Respir Viruses. 2024 Jan 29;18(1):e13252. doi: 10.1111/irv.13252 (PMC10824601; doi:10.1111/irv.13252)
Supplement: Supplementary file 1 — Table S1. List of Primers for Multiplex RT‐PCR Amplification of Influenza A and B Viruses Table S2. List of accession number (GenBank) of each segment of studied influenza viruses Table S3. List of Reference Sequences Downloaded from GISAID Table S4. Summary Table of Average Number of Mapped Reads and Average Coverage Depth for Study Samples Table S5. Distribution of influenza virus subtypes by age groups from our study specimens between July and December of 2019 in NSW, Australia Table S6. Identification of amino acid mutations associated with antiviral drug (NAIs) resistance ‐ seasonal influenza viruses Table S7. Identification of amino acid mutations related to antiviral drug (M2) resistance ‐ seasonal influenza viruses Table S8. Identification of amino acid mutation related to antiviral drug (Baloxavir) resistance ‐ seasonal influenza viruses Table S9. List of amino acid differences in the internal proteins between NSW 2019 viruses and the vaccine strains in the same season [file IRV-18-e13252-s001.doc]

**Supplementary Material**

**Genome Characterization of Influenza A and B Viruses in New South Wales, Australia in 2019: A Retrospective Study Using High-throughput Whole Genome Sequencing**

Xinye Wang^1,2^, Ki Wook Kim^2,3^, Gregory Walker^1,2^, Sacha Stelzer-Braid^1,2^, Matthew Scotch^4,5,6‡^, William D Rawlinson^1,2‡^

**Affiliations**

^1^ School of Biomedical Sciences, Faculty of Medicine and Health, University of New South Wales, Sydney, NSW, Australia

^2^ Virology Research Laboratory, Serology and Virology Division (SAViD), NSW Health Pathology, Prince of Wales Hospital, Sydney, NSW, Australia.

^3^ Discipline of Paediatrics and Child Health, School of Clinical Medicine, Faculty of Medicine and Health, University of New South Wales, Sydney, NSW, Australia.

^4^ Biodesign Center for Environmental Health Engineering, Biodesign Institute, Arizona State University, Phoenix, Arizona, USA.

^5^ College of Health Solutions, Arizona State University, Phoenix, Arizona, USA.

^6^ Kirby Institute, University of New South Wales, Sydney, New South Wales, Australia.

**Methods**

***Clinical Specimens***

The previous record showed no co-infection with other common respiratory viruses was detected in these archived influenza-positive specimens.

***RNA Extraction and RT-qPCR***

The specific primers and probes for influenza RT-qPCR targeted the M gene of the Influenza A virus and the NS gene of the influenza B virus respectively.

***Full-Genome Amplification and Illumina Sequencing***

The influenza A assay was undertaken using a modified protocol with 5 µL of cDNA, 25 µL of 2x RT-PCR buffer, 1 µL of SuperScript III Platinum Taq High Fidelity DNA Polymerase, 0.4 µL of Uni12/Inf1 (10μM), 0.6 µL of Uni12/Inf3 (10μM), and 1 µL of Uni13/Inf1 (10μM). Thermocycler conditions were: 45 °C for 60 min, 94 °C for 2 min, and then 5 cycles (94 °C for 30s, 44 °C for 30s, and 68 °C for 3 min), followed by 31 cycles (94 °C for 30s, 57 °C for 30s, and 68 °C for 3 min), and a 7 min extension at 68 °C. Amplification of the influenza B genomes was performed in 50 µL reactions containing 25 µL of 2x RT-PCR buffer, 1 µL of the SuperScript III Platinum Taq High Fidelity DNA Polymerase, and 4 µL of the IBV-GA2 universal primer cocktail (10 μM). Thermocycler conditions were: 45 °C for 60 min, 55 °C for 30 min, 94 °C for 2min, and then 5 cycles (94 °C for 20s, 40 °C for 30s, and 68 °C for 3 min 30s), followed by 41 cycles (94 °C for 20s, 58 °C for 30s, and 68 °C for 3 min 30s), and a 10 min extension at 68 °C. PCR products were confirmed on a 1% Tris, borate, and ETDA (TBE) agarose gel.

Prior to sequencing on the MiSeq platform, library fragment sizes were checked using the 4200 TapeStation System (Agilent Technologies, USA) to determine an average expected fragment size of 500-600 bp. During the preparation of the sequencing run, a five percent Phi-X spike-in (Illumina, USA) was added to the libraries to enhance their diversity. Pair-end sequencing was performed on a MiSeq platform using a 300-cycle (2 x 150 bp pair-end) MiSeq reagent kit v2 (Illumina, USA)

**Supplemental Tables/Figures**

All supplemental tables and figures are listed below.

| **Table S1.** List of Primers for Multiplex RT-PCR Amplification of Influenza A and B Viruses    ***Specific Primers for Influenza A Virus*** | |
| --- | --- |
| **Primer Name** | **Seqeunce 5' - 3'** |
| Uni12/Inf-1 | GGGGGGAGCAAAAGCAGG |
| Uni12/Inf-3 | GGGGGGAGCGAAAGCAGG |
| Uni13/Inf-1 | CGGGTTATTAGTAGAAACAAGG |
|  |  |
| ***Specific Primers (Universal IBV-GA2 primer cocktail) for Influenza B Virus*** | |
| **Primer Name** | **Seqeunce 5' - 3'** |
| B-PBs-UniF | GGGGGGAGCAGAAGCGGAGC |
| B-PBs-UniR | CCGGGTTATTAGTAGAAACACGAGC |
| B-PA-UniF | GGGGGGAGCAGAAGCGGTGC |
| B-PA-UniR | CCGGGTTATTAGTAGAAACACGTGC |
| B-HANA-UniF | GGGGGGAGCAGAAGCAGAGC |
| B-HANA-UniR | CCGGGTTATTAGTAGTAACAAGAGC |
| B-NP-UniF | GGGGGGAGCAGAAGCACAGC |
| B-NP-UniR | CCGGGTTATTAGTAGAAACAACAGC |
| B-M-Uni3F | GGGGGGAGCAGAAGCACGCACTT |
| B-Mg-Uni3F | GGGGGGAGCAGAAGCAGGCACTT |
| B-M-Uni3R | CCGGGTTATTAGTAGAAACAACGCACTT |
| B-NS-Uni3F | GGGGGGAGCAGAAGCAGAGGATT |
| B-NS-Uni3R | CCGGGTTATTAGTAGTAACAAGAGGATT |

**Table S2.** List of accession number (GenBank) of each segment of studied influenza viruses

|  | **Accession ID number** | | | | | | | |
| --- | --- | --- | --- | --- | --- | --- | --- | --- |
| **Sample ID (S)** | **PB2** | **PB1** | **PA** | **HA** | **NP** | **NA** | **MP** | **NS** |
| *A/H3N2* | | | | | | | | |
| 1 | OQ652817 | OQ652908 | OQ653135 | OQ587837 | OQ652701 | OQ652154 | OQ652587 | OQ653251 |
| 2 | OQ652818 | OQ652909 | OQ653149 | OQ587851 | OQ652702 | OQ652168 | OQ652588 | OQ653252 |
| 3 | OQ652819 | OQ652910 | OQ653157 | OQ587859 | OQ652703 | OQ652176 | OQ652589 | OQ653253 |
| 4 | OQ652820 | OQ652911 | OQ653168 | OQ587870 | OQ652704 | OQ652187 | OQ652590 | OQ653254 |
| 5 | OQ652821 | OQ652912 | OQ653178 | OQ587880 | OQ652705 | OQ652197 | OQ652591 | OQ653255 |
| 6 | OQ652822 | OQ652913 | OQ653185 | OQ587887 | OQ652706 | OQ652204 | OQ652592 | OQ653256 |
| 7 | OQ652823 | OQ652914 | OQ653191 | OQ587894 | OQ652707 | OQ652211 | OQ652593 | OQ653257 |
| 8 | OQ652824 | OQ652915 | OQ653192 | OQ587895 | OQ652708 | OQ652212 | OQ652594 | OQ653258 |
| 9 | OQ652825 | OQ652916 | OQ653196 | OQ587899 | OQ652709 | OQ652216 | OQ652595 | OQ653259 |
| 10 | OQ652826 | OQ652917 | OQ653136 | OQ587838 | OQ652710 | OQ652155 | OQ652596 | OQ653260 |
| 11 | OQ652827 | OQ652918 | OQ653140 | OQ587842 | OQ652711 | OQ652159 | OQ652597 | OQ653261 |
| 12 | OQ652828 | OQ652919 | OQ653141 | OQ587843 | OQ652712 | OQ652160 | OQ652598 | OQ653262 |
| 13 | OQ652829 | OQ652920 | OQ653142 | OQ587844 | OQ652713 | OQ652161 | OQ652599 | OQ653263 |
| 14 | OQ652830 | OQ652921 | OQ653143 | OQ587845 | OQ652714 | OQ652162 | OQ652600 | OQ653264 |
| 15 | OQ652831 | OQ652922 | OQ653144 | OQ587846 | OQ652715 | OQ652163 | OQ652601 | OQ653265 |
| 16 | OQ652832 | OQ652923 | OQ653145 | OQ587847 | OQ652716 | OQ652164 | OQ652602 | OQ653266 |
| 17 | OQ652833 | OQ652924 | OQ653146 | OQ587848 | OQ652717 | OQ652165 | OQ652603 | OQ653267 |
| 18 | OQ652834 | OQ652925 | OQ653147 | OQ587849 | OQ652718 | OQ652166 | OQ652604 | OQ653268 |
| 19 | OQ652835 | OQ652926 | OQ653148 | OQ587850 | OQ652719 | OQ652167 | OQ652605 | OQ653269 |
| 20 | OQ652836 | OQ652927 | OQ653150 | OQ587852 | OQ652720 | OQ652169 | OQ652606 | OQ653270 |
| 22 | OQ652837 | OQ652928 | OQ653151 | OQ587853 | OQ652721 | OQ652170 | OQ652607 | OQ653271 |
| 23 | OQ652857 | OQ652929 | OQ653152 | OQ587854 | OQ652722 | OQ652171 | OQ652608 | OQ653291 |
| 25 | OQ652858 | OQ652930 | OQ653153 | OQ587855 | OQ652723 | OQ652172 | OQ652609 | OQ653311 |
| 27 | OQ652859 | OQ652931 | OQ653154 | OQ587856 | OQ652724 | OQ652173 | OQ652610 | OQ653318 |
| 28 | OQ652860 | OQ652933 | OQ653155 | OQ587857 | OQ652725 | OQ652174 | OQ652611 | OQ653319 |
| 29 | OQ652861 | OQ652932 | OQ653156 | OQ587858 | OQ652726 | OQ652175 | OQ652612 | OQ653320 |
| 30 | OQ652862 | OQ652934 | OQ653158 | OQ587860 | OQ652727 | OQ652177 | OQ652613 | OQ653321 |
| 31 | OQ652863 | OQ652935 | OQ653159 | OQ587861 | OQ652728 | OQ652178 | OQ652614 | OQ653322 |
| 32 | OQ652864 | OQ652936 | OQ653160 | OQ587862 | OQ652729 | OQ652179 | OQ652615 | OQ653292 |
| 33 | OQ652865 | OQ652937 | OQ653161 | OQ587863 | OQ652730 | OQ652180 | OQ652616 | OQ653293 |
| 34 | OQ652866 | OQ652938 | OQ653162 | OQ587864 | OQ652731 | OQ652181 | OQ652617 | OQ653294 |
| 35 | OQ652867 | OQ652939 | OQ653163 | OQ587865 | OQ652732 | OQ652182 | OQ652618 | OQ653295 |
| 36 | OQ652868 | OQ652940 | OQ653164 | OQ587866 | OQ652733 | OQ652183 | OQ652619 | OQ653296 |
| 37 | OQ652869 | OQ652941 | OQ653165 | OQ587867 | OQ652734 | OQ652184 | OQ652620 | OQ653297 |
| 38 | OQ652870 | OQ652942 | OQ653166 | OQ587868 | OQ652735 | OQ652185 | OQ652621 | OQ653298 |
| 39 | OQ652871 | OQ652943 | OQ653167 | OQ587869 | OQ652736 | OQ652186 | OQ652622 | OQ653299 |
| 40 | OQ652872 | OQ652944 | OQ653169 | OQ587871 | OQ652737 | OQ652188 | OQ652623 | OQ653300 |
| 41 | OQ652873 | OQ652945 | OQ653170 | OQ587872 | OQ652738 | OQ652189 | OQ652624 | OQ653301 |
| 42 | OQ652874 | OQ652946 | OQ653171 | OQ587873 | OQ652739 | OQ652190 | OQ652625 | OQ653302 |
| 44 | OQ652875 | OQ652947 | OQ653172 | OQ587874 | OQ652740 | OQ652191 | OQ652626 | OQ653303 |
| 45 | OQ652876 | OQ652948 | OQ653173 | OQ587875 | OQ652741 | OQ652192 | OQ652627 | OQ653304 |
| 46 | OQ652877 | OQ652949 | OQ653174 | OQ587876 | OQ652742 | OQ652193 | OQ652628 | OQ653305 |
| 47 | OQ652878 | OQ652950 | OQ653175 | OQ587877 | OQ652743 | OQ652194 | OQ652629 | OQ653306 |
| 48 | OQ652879 | OQ652951 | OQ653176 | OQ587878 | OQ652744 | OQ652195 | OQ652630 | OQ653307 |
| 49 | OQ652880 | OQ652952 | OQ653177 | OQ587879 | OQ652745 | OQ652196 | OQ652631 | OQ653308 |
| 50 | OQ652881 | OQ652953 | OQ653179 | OQ587881 | OQ652746 | OQ652198 | OQ652632 | OQ653309 |
| 51 | OQ652882 | OQ652954 | OQ653180 | OQ587882 | OQ652747 | OQ652199 | OQ652633 | OQ653310 |
| 53 | OQ652883 | OQ652955 | OQ653181 | OQ587883 | OQ652748 | OQ652200 | OQ652634 | OQ653312 |
| 55 | OQ652838 | OQ652956 | OQ653182 | OQ587884 | OQ652749 | OQ652201 | OQ652635 | OQ653272 |
| 56 | OQ652884 | OQ652957 | OQ653183 | OQ587885 | OQ652750 | OQ652202 | OQ652636 | OQ653313 |
| 59 | OQ652885 | OQ652958 | OQ653184 | OQ587886 | OQ652751 | OQ652203 | OQ652637 | OQ653314 |
| 60 | - | - | - | OQ587888 | OQ652752 | OQ652205 | OQ652638 | OQ653315 |
| 61 | OQ652886 | OQ652959 | OQ653186 | OQ587889 | OQ652753 | OQ652206 | OQ652639 | OQ653316 |
| 62 | OQ652887 | OQ652960 | OQ653187 | OQ587890 | OQ652754 | OQ652207 | OQ652640 | OQ653317 |
| 63 | OQ652839 | OQ652961 | OQ653188 | OQ587891 | OQ652755 | OQ652208 | OQ652641 | OQ653275 |
| 64 | OQ652840 | OQ652962 | OQ653189 | OQ587892 | OQ652756 | OQ652209 | OQ652642 | OQ653285 |
| 66 | OQ652841 | OQ652963 | OQ653190 | OQ587893 | OQ652757 | OQ652210 | OQ652643 | OQ653290 |
| 87 | OQ652842 | OQ652964 | OQ653193 | OQ587896 | OQ652758 | OQ652213 | OQ652644 | OQ653273 |
| 88 | OQ652843 | OQ652965 | OQ653194 | OQ587897 | OQ652759 | OQ652214 | OQ652645 | OQ653274 |
| 89 | OQ652844 | OQ652966 | OQ653195 | OQ587898 | OQ652760 | OQ652215 | OQ652646 | OQ653276 |
| 90 | OQ652845 | OQ652967 | OQ653197 | OQ587900 | OQ652761 | OQ652217 | OQ652647 | OQ653277 |
| 91 | OQ652846 | OQ652968 | OQ653198 | OQ587901 | OQ652762 | OQ652218 | OQ652648 | OQ653278 |
| 92 | OQ652847 | OQ652969 | OQ653199 | OQ587902 | OQ652763 | OQ652219 | OQ652649 | OQ653279 |
| 93 | OQ652848 | OQ652970 | OQ653200 | OQ587903 | OQ652764 | OQ652220 | OQ652650 | OQ653280 |
| 94 | OQ652849 | OQ652971 | OQ653201 | OQ587904 | OQ652765 | OQ652221 | OQ652651 | OQ653281 |
| 95 | OQ652850 | OQ652972 | OQ653202 | OQ587905 | OQ652766 | OQ652222 | OQ652652 | OQ653282 |
| 97 | OQ652851 | OQ652973 | OQ653203 | OQ587906 | OQ652767 | OQ652223 | OQ652653 | OQ653283 |
| 98 | OQ652852 | OQ652974 | OQ653204 | OQ587907 | OQ652768 | OQ652224 | OQ652654 | OQ653284 |
| 99 | OQ652853 | OQ652975 | OQ653205 | OQ587908 | OQ652769 | OQ652225 | OQ652655 | OQ653286 |
| 100 | OQ652854 | OQ652976 | OQ653137 | OQ587839 | OQ652770 | OQ652156 | OQ652656 | OQ653287 |
| 101 | OQ652855 | OQ652977 | OQ653138 | OQ587840 | OQ652771 | OQ652157 | OQ652657 | OQ653288 |
| 102 | OQ652856 | OQ652978 | OQ653139 | OQ587841 | OQ652772 | OQ652158 | OQ652658 | OQ653289 |
| *A/H1N1pdm09* | | | | | | | | |
| 21 | OQ652888 | OQ652979 | OQ653206 | OQ594026 | OQ652792 | OQ652226 | OQ652659 | OQ653323 |
| 24 | OQ652889 | OQ652980 | OQ653220 | OQ594027 | OQ652778 | OQ652227 | OQ652660 | OQ653324 |
| 43 | OQ652891 | OQ652981 | OQ653221 | OQ594028 | OQ652777 | OQ652228 | OQ652661 | OQ653325 |
| 52 | OQ652892 | OQ652982 | OQ653222 | OQ594029 | OQ652776 | OQ652229 | OQ652662 | OQ653326 |
| 54 | OQ652893 | OQ652983 | OQ653223 | OQ594030 | OQ652775 | OQ652230 | OQ652663 | OQ653327 |
| 57 | OQ652894 | OQ652984 | OQ653224 | OQ594031 | OQ652774 | OQ652231 | OQ652664 | OQ653328 |
| 58 | OQ652895 | OQ652985 | OQ653225 | OQ594032 | OQ652773 | OQ652232 | OQ652665 | OQ653329 |
| 65 | OQ652896 | OQ652986 | OQ653214 | OQ594033 | OQ652784 | OQ652233 | OQ652666 | OQ653330 |
| 67 | OQ652897 | OQ652987 | OQ653216 | OQ594034 | OQ652782 | OQ652234 | OQ652667 | OQ653331 |
| 68 | OQ652898 | OQ652988 | OQ653217 | OQ594035 | OQ652781 | OQ652235 | OQ652668 | OQ653332 |
| 69 | OQ652899 | OQ652989 | OQ653218 | OQ594036 | OQ652780 | OQ652236 | OQ652669 | OQ653333 |
| 70 | OQ652900 | OQ652990 | OQ653219 | OQ594037 | OQ652779 | OQ652237 | OQ652670 | OQ653334 |
| 96 | OQ652890 | OQ652991 | OQ653207 | OQ594038 | OQ652791 | OQ652238 | OQ652671 | OQ653335 |
| 103 | OQ652901 | OQ652992 | OQ653208 | OQ594039 | OQ652790 | OQ652239 | OQ652672 | OQ653336 |
| 104 | OQ652902 | OQ652993 | OQ653209 | OQ594040 | OQ652789 | OQ652240 | OQ652673 | OQ653337 |
| 105 | OQ652903 | OQ652994 | OQ653210 | OQ594041 | OQ652788 | OQ652241 | OQ652674 | OQ653338 |
| 106 | OQ652904 | OQ652995 | OQ653211 | OQ594042 | OQ652787 | OQ652242 | OQ652675 | OQ653339 |
| 107 | OQ652905 | OQ652996 | OQ653212 | OQ594043 | OQ652786 | OQ652243 | OQ652676 | OQ653340 |
| 108 | OQ652906 | OQ652997 | OQ653213 | OQ594044 | OQ652785 | OQ652244 | OQ652677 | OQ653341 |
| 109 | OQ652907 | OQ652928 | OQ653215 | OQ594045 | OQ652783 | OQ652245 | OQ652678 | OQ653342 |
| *B/Victoria lineage* | | | | | | | | |
| 71 | OQ652913 | OQ653110 | OQ653239 | OQ594369 | OQ652798 | OQ652246 | OQ652679 | OQ653350 |
| 72 | OQ652914 | OQ653111 | OQ653240 | OQ594370 | OQ652799 | OQ652247 | OQ652680 | OQ653351 |
| 73 | OQ652915 | OQ653112 | OQ653241 | OQ594371 | OQ652800 | OQ652248 | OQ652681 | OQ653352 |
| 74 | OQ652916 | OQ653113 | OQ653242 | OQ594372 | OQ652801 | OQ652249 | OQ652682 | OQ653353 |
| 75 | OQ652917 | OQ653114 | OQ653243 | OQ594373 | OQ652802 | OQ652250 | OQ652683 | OQ653354 |
| 76 | OQ652918 | OQ653115 | OQ653244 | OQ594374 | OQ652803 | OQ652251 | OQ652684 | OQ653355 |
| 77 | OQ652919 | OQ653116 | OQ653245 | OQ594375 | OQ652804 | OQ652252 | OQ652685 | OQ653356 |
| 78 | OQ652920 | OQ653117 | OQ653246 | OQ594376 | OQ652805 | OQ652253 | OQ652686 | OQ653357 |
| 79 | OQ652921 | OQ653118 | OQ653226 | OQ594377 | OQ652806 | OQ652254 | OQ652687 | OQ653358 |
| 80 | OQ652922 | OQ653119 | OQ653227 | OQ594378 | OQ652807 | OQ652255 | OQ652688 | OQ653359 |
| 81 | OQ652923 | OQ653120 | OQ653228 | OQ594379 | OQ652808 | OQ652256 | OQ652689 | OQ653360 |
| 82 | OQ652924 | OQ653121 | OQ653229 | OQ594380 | OQ652809 | OQ652257 | OQ652690 | OQ653361 |
| 83 | OQ652925 | OQ653122 | OQ653230 | OQ594381 | OQ652810 | OQ652258 | OQ652691 | OQ653362 |
| 84 | OQ652926 | OQ653123 | OQ653231 | OQ594382 | OQ652811 | OQ652259 | OQ652692 | OQ653363 |
| 85 | OQ652927 | OQ653124 | OQ653232 | OQ594383 | OQ652812 | OQ652260 | OQ652693 | OQ653364 |
| 86 | OQ652928 | OQ653125 | OQ653233 | OQ594384 | OQ652813 | OQ652261 | OQ652694 | OQ653365 |
| 110 | OQ652908 | OQ653126 | OQ653234 | OQ594385 | OQ652793 | OQ652262 | OQ652695 | OQ653366 |
| 111 | OQ652909 | OQ653127 | OQ653235 | OQ594386 | OQ652794 | OQ652263 | OQ652696 | OQ653367 |
| 112 | OQ652910 | OQ653128 | OQ653236 | OQ594387 | OQ652795 | OQ652264 | OQ652697 | OQ653368 |
| 113 | OQ652911 | OQ653129 | OQ653237 | OQ594388 | OQ652796 | OQ652265 | OQ652698 | OQ653369 |
| 114 | OQ652912 | OQ653130 | OQ653238 | OQ594389 | OQ652797 | OQ652266 | OQ652699 | OQ653370 |

**Table S3.** List of Reference Sequences Downloaded from GISAID

| **Strain Name** | **Accession ID** | **Segment** | **Accession ID** | **Segment** | **Date (DD/MM/YYYY)** |
| --- | --- | --- | --- | --- | --- |
| ***A/H3N2 viruses*** |  |  |  |  |  |
| A/Switzerland/8060/2017 | EPI2397203 | HA | EPI2397205 | NA | 2017 |
| A/Texas/50/2012 | EPI2397064 | HA | EPI2397066 | NA | 2012 |
| A/Victoria/2574/2019 | EPI1647443 | HA | EPI1647442 | NA | 12/11/2019 |
| A/Victoria/268/2019 | EPI1658617 | HA | EPI1658616 | NA | 23/10/2019 |
| A/Victoria/2833/2019 | EPI1671630 | HA | EPI1671629 | NA | 21/9/2019 |
| A/Victoria/151/2019 | EPI1503481 | HA | EPI1503480 | NA | 19/7/2019 |
| A/Victoria/93/2019 | EPI1670138 | HA | EPI1670137 | NA | 12/6/2019 |
| A/Victoria/120/2019 | [EPI1670150](https://platform.epicov.org/epi3/start/EPI/1670150) | HA | EPI1670149 | NA | 24/6/2019 |
| A/Victoria/2690/2019 | [EPI1670198](https://platform.epicov.org/epi3/start/EPI/1670198) | HA | EPI1670197 | NA | 21/6/2019 |
| A/SouthAustralia/472/2019 | EPI1647467 | HA | EPI1647466 | NA | 23/11/2019 |
| A/SouthAustralia/458/2019 | EPI1658832 | HA | EPI1658831 | NA | 31/10/2019 |
| A/SouthAustralia/389/2019 | EPI1740036 | HA | EPI1740035 | NA | 10/7/2019 |
| A/SouthAustralia/1126/2019 | EPI1740100 | HA | EPI1740099 | NA | 22/8/2019 |
| A/SouthAustralia/1088/2019 | EPI1670105 | HA | EPI1670104 | NA | 30/5/2019 |
| A/SouthAustralia/369/2019 | EPI1740121 | HA | EPI1740120 | NA | 13/6/2019 |
| A/Tasmania/1037/2019 | EPI1647915 | HA | EPI1647914 | NA | 30/9/2019 |
| A/Tasmania/1038/2019 | EPI1658827 | HA | EPI1658826 | NA | 24/10/2019 |
| A/Tasmania/650/2019 | EPI1690298 | HA | EPI1690297 | NA | 16/11/2019 |
| A/Tasmania/76/2019 | EPI1658779 | HA | EPI1658778 | NA | 6/8/2019 |
| A/Tasmania/1014/2019 | EPI1589616 | HA | EPI1589615 | NA | 28/6/2019 |
| A/Tasmania/49/2019 | EPI1590809 | HA | EPI1590808 | NA | 25/5/2019 |
| A/Tasmania/568/2019 | EPI1671464 | HA | EPI1671463 | NA | 9/6/2019 |
| A/Townsville/59/2019 | EPI1658650 | HA | EPI1658649 | NA | 18/10/2019 |
| A/Brisbane/141/2019 | EPI1658812 | HA | EPI1658811 | NA | 6/10/2019 |
| A/Brisbane/1030/2019 | EPI1667407 | HA | EPI1667406 | NA | 8/7/2019 |
| A/Townsville/48/2019 | EPI1671531 | HA | EPI1671530 | NA | 12/8/2019 |
| A/Brisbane/33/2019 | EPI1590836 | HA | EPI1590835 | NA | 10/5/2019 |
| A/Brisbane/67/2019 | EPI1671480 | HA | EPI1671479 | NA | 29/6/2019 |
| A/Perth/197/2019 | EPI1740044 | HA | EPI1740043 | NA | 15/10/2019 |
| A/Perth/134/2019 | EPI1670070 | HA | EPI1670069 | NA | 2/7/2019 |
| A/Perth/1131/2019 | EPI1667399 | HA | EPI1667398 | NA | 8/7/2019 |
| A/Perth/1127/2019 | EPI1589588 | HA | EPI1589587 | NA | 4/7/2019 |
| A/Perth/103/2019 | EPI1670025 | HA | EPI1670024 | NA | 9/6/2019 |
| A/Perth/1056/2019 | EPI1670231 | HA | EPI1670230 | NA | 5/6/2019 |
| A/Perth/117/2019 | EPI1671489 | HA | EPI1671488 | NA | 17/6/2019 |
| A/Canberra/398/2019 | EPI1671959 | HA | EPI1671958 | NA | 2/10/2019 |
| A/Canberra/399/2019 | EPI1740052 | HA | EPI1740051 | NA | 4/10/2019 |
| A/Canberra/407/2019 | EPI1713737 | HA | EPI1713736 | NA | 21/10/2019 |
| A/Canberra/2A/2019 | EPI1690280 | HA | EPI1690279 | NA | 1/12/2019 |
| A/Canberra/213/2019 | EPI1590857 | HA | EPI1590856 | NA | 5/6/2019 |
| A/Canberra/187/2019 | EPI1665107 | HA | EPI1665106 | NA | 27/5/2019 |
| A/Canberra/1006/2019 | EPI1667303 | HA | EPI1667302 | NA | 21/6/2019 |
| A/Darwin/726/2019 | EPI1696166 | HA | EPI1696165 | NA | 3/10/2019 |
| A/Darwin/845/2019 | EPI1690259 | HA | EPI1690258 | NA | 24/12/2019 |
| A/Darwin/610/2019 | EPI1670040 | HA | EPI1670039 | NA | 25/7/2019 |
| A/Darwin/552/2019 | EPI1583466 | HA | EPI1583465 | NA | 1/7/2019 |
| A/Darwin/423/2019 | [EPI1665130](https://platform.epicov.org/epi3/start/EPI/1665130) | HA | [EPI1665129](https://platform.epicov.org/epi3/start/EPI/1665129) | NA | 17/5/2019 |
| A/Darwin/1010/2019 | [EPI1670225](https://platform.epicov.org/epi3/start/EPI/1670225) | HA | [EPI1670224](https://platform.epicov.org/epi3/start/EPI/1670224) | NA | 3/6/2019 |
| A/Darwin/542/2019 | [EPI1670240](https://platform.epicov.org/epi3/start/EPI/1670240) | HA | [EPI1670239](https://platform.epicov.org/epi3/start/EPI/1670239) | NA | 27/6/2019 |
| A/Wellington/52/2019 | [EPI1671504](https://platform.epicov.org/epi3/start/EPI/1671504) | HA | [EPI1671503](https://platform.epicov.org/epi3/start/EPI/1671503) | NA | 23/8/2019 |
| A/Wellington/53/2019 | [EPI1671507](https://platform.epicov.org/epi3/start/EPI/1671507) | HA | [EPI1671506](https://platform.epicov.org/epi3/start/EPI/1671506) | NA | 23/9/2019 |
| A/Canterbury/31/2019 | [EPI1671510](https://platform.epicov.org/epi3/start/EPI/1671510) | HA | [EPI1671509](https://platform.epicov.org/epi3/start/EPI/1671509) | NA | 2/7/2019 |
| A/Canterbury/33/2019 | [EPI1671513](https://platform.epicov.org/epi3/start/EPI/1671513) | HA | [EPI1671512](https://platform.epicov.org/epi3/start/EPI/1671512) | NA | 13/8/2019 |
| A/Christchurch/519/2019 | [EPI1583929](https://platform.epicov.org/epi3/start/EPI/1583929) | HA | [EPI1583928](https://platform.epicov.org/epi3/start/EPI/1583928) | NA | 4/6/2019 |
| A/Wellington/27/2019 | [EPI1583982](https://platform.epicov.org/epi3/start/EPI/1583982) | HA | [EPI1583981](https://platform.epicov.org/epi3/start/EPI/1583981) | NA | 20/5/2019 |
| A/Christchurch/529/2019 | [EPI1590902](https://platform.epicov.org/epi3/start/EPI/1590902) | HA | [EPI1590901](https://platform.epicov.org/epi3/start/EPI/1590901) | NA | 27/5/2019 |
| A/Christchurch/528/2019 | [EPI1590905](https://platform.epicov.org/epi3/start/EPI/1590905) | HA | [EPI1590904](https://platform.epicov.org/epi3/start/EPI/1590904) | NA | 27/5/2019 |
| A/Qatar/16-VI-19-0049409/2019 | EPI1802146 | HA | EPI1802145 | NA | 27/8/2019 |
| A/Pennsylvania/1026/2019 | EPI1743962 | HA | EPI1743961 | NA | 4/11/2019 |
| A/England/767/2019 | EPI1741245 | HA | EPI1741244 | NA | 16/12/2019 |
| A/Texas/71/2017 | EPI973255 | HA | EPI973254 | NA | 18/3/2017 |
|  |  |  |  |  |  |
| ***A/H1N1pdm09 viruses*** |  |  |  |  |  |
| A/California/07/2009 | EPI516535 | HA | EPI516534 | NA | 9/4/2009 |
| A/Michigan/45/2015 | EPI685579 | HA | EPI685578 | NA | 7/9/2015 |
| A/Victoria/244/2019 | EPI1618343 | HA | EPI1618342 | NA | 2/9/2019 |
| A/Victoria/2455/2019 | EPI1647304 | HA | EPI1647303 | NA | 28/8/2019 |
| A/Victoria/2570/2019 | EPI1813675 | HA | EPI1813674 | NA | 22/11/2019 |
| A/Victoria/267/2019 | EPI1647865 | HA | EPI1647864 | NA | 17/10/2019 |
| A/Victoria/2605/2019 | EPI1690358 | HA | EPI1690357 | NA | 11/12/2019 |
| A/Victoria/107/2019 | EPI1594186 | HA | EPI1594185 | NA | 18/6/2019 |
| A/Victoria/761/2019 | EPI1665055 | HA | EPI1665054 | NA | 18/5/2019 |
| A/Brisbane/147/2019 | EPI1647880 | HA | EPI1647879 | NA | 24/10/2019 |
| A/Brisbane/90/2019 | EPI1647838 | HA | EPI1647837 | NA | 29/7/2019 |
| A/Brisbane/1127/2019 | EPI1718516 | HA | EPI1718515 | NA | 13/9/2019 |
| A/Brisbane/102/2019 | EPI1589552 | HA | EPI1589551 | NA | 15/8/2019 |
| A/Brisbane/66/2019 | EPI1584512 | HA | EPI1584511 | NA | 3/7/2019 |
| A/Brisbane/43/2019 | [EPI1594246](https://platform.epicov.org/epi3/start/EPI/1594246) | HA | [EPI1594245](https://platform.epicov.org/epi3/start/EPI/1594245) | NA | 22/5/2019 |
| A/Brisbane/1025/2019 | [EPI1647168](https://platform.epicov.org/epi3/start/EPI/1647168) | HA | [EPI1647167](https://platform.epicov.org/epi3/start/EPI/1647167) | NA | 27/6/2019 |
| A/Brisbane/61/2019 | [EPI1647280](https://platform.epicov.org/epi3/start/EPI/1647280) | HA | [EPI1647279](https://platform.epicov.org/epi3/start/EPI/1647279) | NA | 18/6/2019 |
| A/SouthAustralia/402/2019 | EPI1721604 | HA | EPI1721603 | NA | 30/7/2019 |
| A/SouthAustralia/1128/2019 | EPI1647566 | HA | EPI1647565 | NA | 2/12/2019 |
| A/SouthAustralia/456/2019 | EPI1647300 | HA | EPI1647299 | NA | 31/10/2019 |
| A/SouthAustralia/473/2019 | EPI1647575 | HA | EPI1647574 | NA | 29/11/2019 |
| A/SouthAustralia/1065/2019 | EPI1594102 | HA | EPI1594101 | NA | 8/5/2019 |
| A/Perth/180/2019 | EPI1796540 | HA | EPI1796539 | NA | 3/12/2019 |
| A/Perth/193/2019 | EPI1672613 | HA | EPI1672612 | NA | 12/11/2019 |
| A/Perth/1154/2019 | EPI1647912 | HA | EPI1647911 | NA | 30/10/2019 |
| A/Perth/178/2019 | [EPI1671884](https://platform.epicov.org/epi3/start/EPI/1671884) | HA | [EPI1671883](https://platform.epicov.org/epi3/start/EPI/1671883) | NA | 29/11/2019 |
| A/Perth/195/2019 | [EPI1721596](https://platform.epicov.org/epi3/start/EPI/1721596) | HA | [EPI1721595](https://platform.epicov.org/epi3/start/EPI/1721595) | NA | 28/11/2019 |
| A/Perth/99/2019 | EPI1594297 | HA | EPI1594296 | NA | 27/5/2019 |
| A/Tasmania/604/2019 | EPI1721644 | HA | EPI1721643 | NA | 22/8/2019 |
| A/Tasmania/1017/2019 | EPI1584518 | HA | EPI1584517 | NA | 4/7/2019 |
| A/Tasmania/1010/2019 | [EPI1594099](https://platform.epicov.org/epi3/start/EPI/1594099) | HA | [EPI1594098](https://platform.epicov.org/epi3/start/EPI/1594098) | NA | 6/5/2019 |
| A/Tasmania/1012/2019 | [EPI1594111](https://platform.epicov.org/epi3/start/EPI/1594111) | HA | [EPI1594110](https://platform.epicov.org/epi3/start/EPI/1594110) | NA | 3/6/2019 |
| A/Tasmania/40/2019 | [EPI1594126](https://platform.epicov.org/epi3/start/EPI/1594126) | HA | [EPI1594125](https://platform.epicov.org/epi3/start/EPI/1594125) | NA | 19/5/2019 |
| A/Canberra/337/2019 | EPI1647174 | HA | EPI1647173 | NA | 20/8/2019 |
| A/Canberra/394/2019 | EPI1671732 | HA | EPI1671731 | NA | 1/10/2019 |
| A/Canberra/400/2019 | EPI1671735 | HA | EPI1671734 | NA | 5/10/2019 |
| A/Canberra/397/2019 | EPI1671899 | HA | EPI1671898 | NA | 2/10/2019 |
| A/Canberra/411/2019 | EPI1671908 | HA | EPI1671907 | NA | 3/11/2019 |
| A/Canberra/141/2019 | [EPI1594129](https://platform.epicov.org/epi3/start/EPI/1594129) | HA | [EPI1594128](https://platform.epicov.org/epi3/start/EPI/1594128) | NA | 20/5/2019 |
| A/Canberra/169/2019 | [EPI1665048](https://platform.epicov.org/epi3/start/EPI/1665048) | HA | [EPI1665047](https://platform.epicov.org/epi3/start/EPI/1665047) | NA | 29/5/2019 |
| A/Darwin/639/2019 | EPI1647176 | HA | EPI1647175 | NA | 4/8/2019 |
| A/Darwin/776/2019 | EPI1721620 | HA | EPI1721619 | NA | 1/12/2019 |
| A/Darwin/831/2019 | EPI1690379 | HA | EPI1690378 | NA | 16/12/2019 |
| A/Darwin/324/2019 | [EPI1484482](https://platform.epicov.org/epi3/start/EPI/1484482) | HA | [EPI1484481](https://platform.epicov.org/epi3/start/EPI/1484481) | NA | 7/5/2019 |
| A/Darwin/371/2019 | [EPI1489664](https://platform.epicov.org/epi3/start/EPI/1489664) | HA | [EPI1489663](https://platform.epicov.org/epi3/start/EPI/1489663) | NA | 9/5/2019 |
| A/Darwin/529/2019 | [EPI1594279](https://platform.epicov.org/epi3/start/EPI/1594279) | HA | [EPI1594278](https://platform.epicov.org/epi3/start/EPI/1594278) | NA | 19/6/2019 |
| A/Wellington/36/2019 | EPI1584466 | HA | EPI1584465 | NA | 6/6/2019 |
| A/Christchurch/530/2019 | EPI1594288 | HA | EPI1594287 | NA | 10/6/2019 |
| A/Hawaii/66/2019 | EPI1617919 | HA | EPI1617918 | NA | 19/9/2019 |
| A/Okinawa/93/2019 | EPI1697131 | HA | EPI1697130 | NA | 25/9/2019 |
| A/Nebraska/14/2019 | EPI1619398 | HA | EPI1619397 | NA | 21/10/2019 |
| /Denmark/3280/2019 | EPI1641015 | HA | EPI1641014 | NA | 10/11/2019 |
| A/Iowa/33/2019 | EPI1448830 | HA | EPI1448829 | NA | 3/5/2019 |
| A/Kanagawa/IC1848/2019 | EPI1606763 | HA | EPI1606762 | NA | 18/1/2019 |
|  |  |  |  |  |  |
| ***B/Victoria lineage viruses*** |  |  |  |  |  |
| B/Colorado/06/2017 | EPI1051642 | HA | EPI1051641 | NA | 25/2/2017 |
| B/Brisbane/60/2008 | EPI172555 | HA | EPI172554 | NA | 4/8/2008 |
| B/Victoria/25/2019 | EPI1583300 | HA | EPI1583299 | NA | 1/8/2019 |
| B/Victoria/46/2019 | EPI1647959 | HA | EPI1647958 | NA | 25/9/2019 |
| B/Victoria/2089/2019 | EPI1690449 | HA | EPI1690448 | NA | 2/9/2019 |
| B/Victoria/2108/2019 | EPI1658720 | HA | EPI1658719 | NA | 2/10/2019 |
| B/Victoria/2113/2019 | EPI1733591 | HA | EPI1733590 | NA | 15/11/2019 |
| B/Victoria/704/2019 | EPI1543239 | HA | EPI1543238 | NA | 2/5/2019 |
| B/Victoria/5/2019 | EPI1543263 | HA | EPI1543262 | NA | 27/5/2019 |
| B/Victoria/950/2019 | EPI1584546 | HA | EPI1584545 | NA | 21/6/2019 |
| B/Brisbane/14/2019 | EPI1584422 | HA | EPI1584421 | NA | 14/7/2019 |
| B/Brisbane/27/2019 | EPI1647615 | HA | EPI1647614 | NA | 1/9/2019 |
| B/Brisbane/34/2019 | EPI1658756 | HA | EPI1658755 | NA | 17/9/2019 |
| B/Brisbane/12/2019 | EPI1927050 | HA | EPI1927049 | NA | 8/7/2019 |
| B/Brisbane/21/2019 | EPI1647412 | HA | EPI1647411 | NA | 14/8/2019 |
| B/Brisbane/5/2019 | [EPI1492186](https://platform.epicov.org/epi3/start/EPI/1492186) | HA | [EPI1492185](https://platform.epicov.org/epi3/start/EPI/1492185) | NA | 17/5/2019 |
| B/Brisbane/6/2019 | [EPI1506698](https://platform.epicov.org/epi3/start/EPI/1506698) | HA | [EPI1506697](https://platform.epicov.org/epi3/start/EPI/1506697) | NA | 17/5/2019 |
| B/Brisbane/4/2019 | [EPI1506702](https://platform.epicov.org/epi3/start/EPI/1506702) | HA | [EPI1506701](https://platform.epicov.org/epi3/start/EPI/1506701) | NA | 9/5/2019 |
| B/SouthAustralia/112/2019 | EPI1690447 | HA | EPI1690446 | NA | 16/9/2019 |
| B/SouthAustralia/123/2019 | EPI1647641 | HA | EPI1647640 | NA | 29/9/2019 |
| B/SouthAustralia/122/2019 | EPI1647639 | HA | EPI1647638 | NA | 28/9/2019 |
| B/SouthAustralia/108/2019 | EPI1647593 | HA | EPI1647592 | NA | 4/9/2019 |
| B/SouthAustralia/1022/2019 | EPI1618437 | HA | EPI1618436 | NA | 11/9/2019 |
| B/Perth/60/2019 | EPI1658728 | HA | EPI1658727 | NA | 8/9/2019 |
| B/Perth/61/2019 | EPI1658730 | HA | EPI1658729 | NA | 16/9/2019 |
| B/Perth/80/2019 | EPI1734605 | HA | EPI1734604 | NA | 24/10/2019 |
| B/Perth/48/2019 | EPI1658754 | HA | EPI1658753 | NA | 10/8/2019 |
| B/Perth/21/2019 | [EPI1584528](https://platform.epicov.org/epi3/start/EPI/1584528) | HA | [EPI1584527](https://platform.epicov.org/epi3/start/EPI/1584527) | NA | 10/5/2019 |
| B/Perth/23/2019 | [EPI1584530](https://platform.epicov.org/epi3/start/EPI/1584530) | HA | [EPI1584529](https://platform.epicov.org/epi3/start/EPI/1584529) | NA | 19/5/2019 |
| B/Perth/33/2019 | [EPI1584536](https://platform.epicov.org/epi3/start/EPI/1584536) | HA | [EPI1584535](https://platform.epicov.org/epi3/start/EPI/1584535) | NA | 10/6/2019 |
| B/Tasmania/512/2019 | EPI1647647 | HA | EPI1647646 | NA | 10/9/2019 |
| B/Tasmania/501/2019 | EPI1589996 | HA | EPI1589995 | NA | 6/8/2019 |
| B/Tasmania/500/2019 | EPI1492192 | HA | EPI1492191 | NA | 3/5/2019 |
| B/Canberra/76/2019 | EPI1671727 | HA | EPI1671726 | NA | 22/9/2019 |
| B/Canberra/65/2019 | EPI1647619 | HA | EPI1647618 | NA | 9/9/2019 |
| B/Canberra/55/2019 | EPI1590004 | HA | EPI1590003 | NA | 15/8/2019 |
| B/Canberra/87/2019 | EPI1690413 | HA | EPI1690412 | NA | 14/10/2019 |
| B/Canberra/60/2019 | EPI1647597 | HA | EPI1647596 | NA | 1/9/2019 |
| B/Canberra/42/2019 | EPI1543283 | HA | EPI1543282 | NA | 10/6/2019 |
| B/Canberra/25/2019 | EPI1543279 | HA | EPI1543278 | NA | 4/6/2019 |
| B/Darwin/126/2019 | EPI1647617 | HA | EPI1647616 | NA | 8/9/2019 |
| B/Darwin/176/2019 | EPI1671965 | HA | EPI1671964 | NA | 2/11/2019 |
| B/Darwin/161/2019 | EPI1658712 | HA | EPI1658711 | NA | 16/10/2019 |
| B/Darwin/138/2019 | EPI1647589 | HA | EPI1647588 | NA | 27/9/2019 |
| B/Darwin/146/2019 | EPI1618425 | HA | EPI1618424 | NA | 5/10/2019 |
| B/Darwin/67/2019 | [EPI1492184](https://platform.epicov.org/epi3/start/EPI/1492184) | HA | [EPI1492183](https://platform.epicov.org/epi3/start/EPI/1492183) | NA | 31/5/2019 |
| B/Darwin/55/2019 | [EPI1492196](https://platform.epicov.org/epi3/start/EPI/1492196) | HA | [EPI1492195](https://platform.epicov.org/epi3/start/EPI/1492195) | NA | 4/5/2019 |
| B/Darwin/69/2019 | [EPI1506338](https://platform.epicov.org/epi3/start/EPI/1506338) | HA | [EPI1506337](https://platform.epicov.org/epi3/start/EPI/1506337) | NA | 20/6/2019 |
| B/Darwin/57/2019 | [EPI1543225](https://platform.epicov.org/epi3/start/EPI/1543225) | HA | [EPI1543224](https://platform.epicov.org/epi3/start/EPI/1543224) | NA | 5/5/2019 |
| B/Wellington/106/2019 | EPI1647595 | HA | EPI1647594 | NA | 3/9/2019 |
| B/Wellington/108/2019 | EPI1647601 | HA | EPI1647600 | NA | 11/9/2019 |
| B/Wellington/109/2019 | EPI1647603 | HA | EPI1647602 | NA | 18/9/2019 |
| B/Canterbury/21/2019 | EPI1671961 | HA | EPI1671960 | NA | 12/8/2019 |
| B/Wellington/107/2019 | EPI1671969 | HA | EPI1671968 | NA | 16/9/2019 |
| B/Dunedin/6/2019 | EPI1671971 | HA | EPI1671970 | NA | 9/7/2019 |
| B/Waikato/2/2019 | EPI1671975 | HA | EPI1671974 | NA | 23/7/2019 |
| B/Wellington/111/2019 | [EPI1922874](https://platform.epicov.org/epi3/start/EPI/1922874) | HA | [EPI1922873](https://platform.epicov.org/epi3/start/EPI/1922873) | NA | 23/5/2019 |
| B/Wellington/8/2019 | [EPI1674248](https://platform.epicov.org/epi3/start/EPI/1674248) | HA | [EPI1674247](https://platform.epicov.org/epi3/start/EPI/1674247) | NA | 7/5/2019 |
| B/Christchurch/505/2019 | [EPI1647410](https://platform.epicov.org/epi3/start/EPI/1647410) | HA | [EPI1647409](https://platform.epicov.org/epi3/start/EPI/1647409) | NA | 7/6/2019 |
| B/Dunedin/3/2019 | [EPI1584438](https://platform.epicov.org/epi3/start/EPI/1584438) | HA | [EPI1584437](https://platform.epicov.org/epi3/start/EPI/1584437) | NA | 9/6/2019 |
| B/Wellington/19/2019 | [EPI1584436](https://platform.epicov.org/epi3/start/EPI/1584436) | HA | [EPI1584435](https://platform.epicov.org/epi3/start/EPI/1584435) | NA | 15/5/2019 |
| B/Christchurch/503/2019 | [EPI1543267](https://platform.epicov.org/epi3/start/EPI/1543267) | HA | [EPI1543266](https://platform.epicov.org/epi3/start/EPI/1543266) | NA | 6/5/2019 |
| B/Christchurch/502/2019 | [EPI1543265](https://platform.epicov.org/epi3/start/EPI/1543265) | HA | [EPI1543264](https://platform.epicov.org/epi3/start/EPI/1543264) | NA | 10/6/2019 |
| B/Wellington/4/2019 | [EPI1543257](https://platform.epicov.org/epi3/start/EPI/1543257) | HA | [EPI1543256](https://platform.epicov.org/epi3/start/EPI/1543256) | NA | 2/5/2019 |
| B/Wellington/12/2019 | [EPI1543255](https://platform.epicov.org/epi3/start/EPI/1543255) | HA | [EPI1543254](https://platform.epicov.org/epi3/start/EPI/1543254) | NA | 9/5/2019 |
| B/Wellington/9/2019 | [EPI1543253](https://platform.epicov.org/epi3/start/EPI/1543253) | HA | [EPI1543252](https://platform.epicov.org/epi3/start/EPI/1543252) | NA | 8/5/2019 |
| B/Tauranga/2/2019 | [EPI1506696](https://platform.epicov.org/epi3/start/EPI/1506696) | HA | [EPI1506695](https://platform.epicov.org/epi3/start/EPI/1506695) | NA | 3/5/2019 |
| B/Wellington/16/2019 | [EPI1506692](https://platform.epicov.org/epi3/start/EPI/1506692) | HA | [EPI1506691](https://platform.epicov.org/epi3/start/EPI/1506691) | NA | 17/5/2019 |
| B/Tauranga/1/2019 | [EPI1506688](https://platform.epicov.org/epi3/start/EPI/1506688) | HA | [EPI1506687](https://platform.epicov.org/epi3/start/EPI/1506687) | NA | 5/5/2019 |
| B/Wellington/13/2019 | [EPI1506684](https://platform.epicov.org/epi3/start/EPI/1506684) | HA | [EPI1506683](https://platform.epicov.org/epi3/start/EPI/1506683) | NA | 10/5/2019 |
| B/Christchurch/504/2019 | [EPI1506346](https://platform.epicov.org/epi3/start/EPI/1506346) | HA | [EPI1506345](https://platform.epicov.org/epi3/start/EPI/1506345) | NA | 27/5/2019 |
| B/Canterbury/3/2019 | [EPI1492182](https://platform.epicov.org/epi3/start/EPI/1492182) | HA | [EPI1492181](https://platform.epicov.org/epi3/start/EPI/1492181) | NA | 1/5/2019 |
| B/Wellington/15/2019 | [EPI1492180](https://platform.epicov.org/epi3/start/EPI/1492180) | HA | [EPI1492179](https://platform.epicov.org/epi3/start/EPI/1492179) | NA | 17/5/2019 |
| B/HongKong/574/2019 | EPI1736410 | HA | EPI1736409 | NA | 17/6/2019 |
| B/Maryland/24/2019 | EPI1637818 | HA | EPI1637817 | NA | 6/11/2019 |
| B/Niigata-C/6/2019 | EPI1703596 | HA | EPI1703595 | NA | 27/10/2019 |
| B/Chile/8427/2019 | EPI1665027 | HA | EPI1665026 | NA | 13/11/2019 |

**Table S4.** Summary Table of Average Number of Mapped Reads and Average Coverage Depth for Study Samples

|  |  |  |  |  |  |  |  |  |
| --- | --- | --- | --- | --- | --- | --- | --- | --- |
|  | **Influenza A Virus Genome** | | | | | | | |
|  | ***A/H3N2*** | | | | | | | |
|  | **Segment 1** | **Segment 2** | **Segment 3** | **Segment 4** | **Segment 5** | **Segment 6** | **Segment 7** | **Segment 8** |
|  | **PB2 (2280bp)** | **PB1 (2277bp)** | **PA (2152bp)** | **HA (1702bp)** | **NP (1498bp)** | **NA (1411bp)** | **M** | **NS (836bp)** |
|  |  |  |  |  |  |  | **(983bp)** |  |
| **Average No. of Mapped Reads** | 101700 | 79441 | 106791 | 125195 | 58917 | 37207 | 87763 | 41147 |
| **Average Coverage Depth** | 6429 | 5115 | 7124 | 9909 | 5569 | 3778 | 11345 | 6683 |
|  | ***A/H1N1-pdm09*** | | | | | | | |
|  | **Segment 1** | **Segment 2** | **Segment 3** | **Segment 4** | **Segment 5** | **Segment 6** | **Segment 7** | **Segment 8** |
|  | **PB2 (2280bp)** | **PB1 (2275bp)** | **PA (2151bp)** | **HA (1702bp)** | **NP (1498bp)** | **NA (1411bp)** | **M** | **NS (836bp)** |
|  |  |  |  |  |  |  | **(983bp)** |  |
| **Average No. of Mapped Reads** | 77094 | 54727 | 53755 | 79380 | 76234 | 44798 | 83310 | 98964 |
| **Average Coverage Depth** | 4702 | 3465 | 3575 | 6978 | 7264 | 4569 | 11873 | 14846 |
|  | **Influenza B Virus Genome** | | | | | | | |
|  | ***B/Victoria Lineage*** | | | | | | | |
|  | **Segment 1** | **Segment 2** | **Segment 3** | **Segment 4** | **Segment 5** | **Segment 6** | **Segment 7** | **Segment 8** |
|  | **PB2 (2313bp)** | **PB1 (2260bp)** | **PA (2181bp)** | **HA (1753bp)** | **NP (1684bp)** | **NA (1409bp)** | **M** | **NS (1028bp)** |
|  |  |  |  |  |  |  | **(1077bp)** |  |
| **Average No. of Mapped Reads** | 70862 | 69987 | 53255 | 87127 | 16140 | 73643 | 40558 | 4725 |
| **Average Coverage Depth** | 4319 | 4418 | 3488 | 7210 | 1338 | 7532 | 5221 | 660 |

Notes: PB2 (polymerase basic 2), PB1 (polymerase basic 1), PA (polymerase acid), HA (hemagglutinin), NP (nucleoprotein), NA (neuraminidase), M (matrix), NS (non-structural). All numbers are rounded to integers.

**Table S5.** Distribution of influenza virus subtypes by age groups from our study specimens between July and December of 2019 in NSW, Australia

|  | **Influenza Subtype** | | |  |
| --- | --- | --- | --- | --- |
|  | **A/H3N2 (n=72)** | **A/H1N1pdm09 (n=20)** | **B/Victoria (n=21)** |  |
|  |
| **Age Group** |  |  |  |  |
| **<5** | 9 (56.3%) | 5 (31.3%) | 2 (12.4%) |  |
| **5-17** | 5 (35.7%) | 3 (21.4%) | 6 (42.9%) |  |
| **18-49** | 14 (45.2%) | 6 (19.4%) | 11 (35.4%) |  |
| **50-64** | 9 (69.2%) | 2 (15.4%) | 2 (15.4%) |  |
| **>=65** | 35 (89.7%) | 4 (10.3%) | 0 (0.0%) |  |

**Table S6.** Identification of amino acid mutations associated with antiviral drug (NAIs) resistance - seasonal influenza viruses.


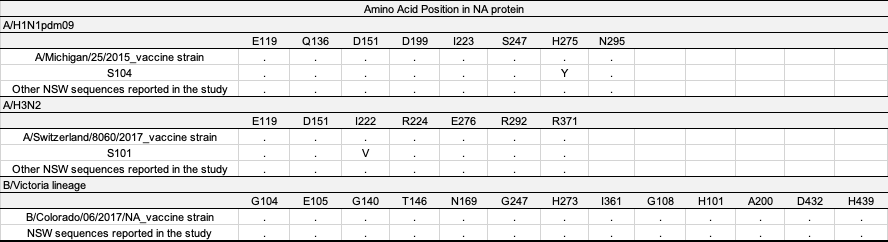


**Table S7**. Identification of amino acid mutations related to antiviral drug (M2) resistance - seasonal influenza viruses.


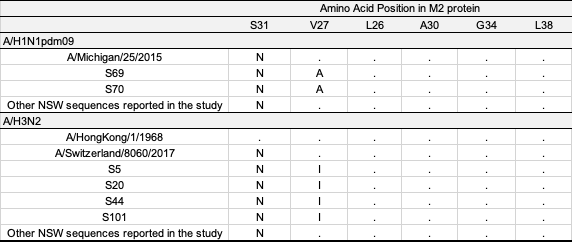


**Table S8.** Identification of amino acid mutation related to antiviral drug (baloxavir) resistance - seasonal influenza viruses.


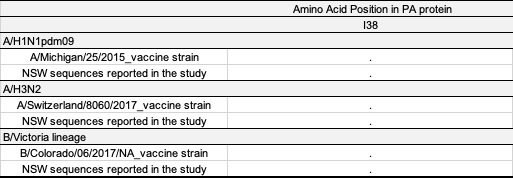


**Table S9**. List of amino acid differences in the internal proteins between NSW 2019 viruses and the vaccine strains in the same season


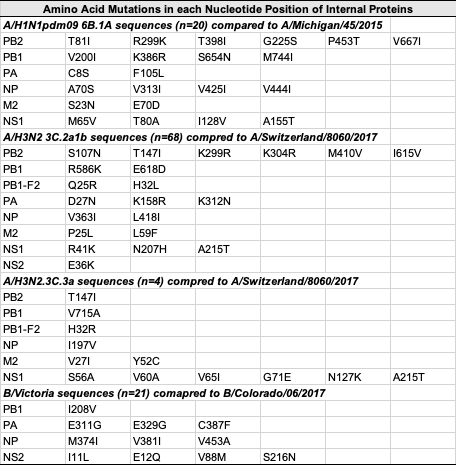


Notes:

| 1. Only amino acid substitutions found in more than two sequences in each genetic clade are presented in this table. 2. Please also note that IBVs do not contain the PB1-F2 protein. 3. All sequences detected in Yr2019. |
| --- |
